# A scoping review of the literature on the application and usefulness of the Problem Management Plus (PM+) intervention around the world

**DOI:** 10.1192/bjo.2024.55

**Published:** 2024-04-23

**Authors:** Patrick N. Mwangala, Millicent Makandi, Anita Kerubo, Moses K. Nyongesa, Amina Abubakar

**Affiliations:** Institute for Human Development, Aga Khan University, Kenya; Centre for Geographic Medicine Research Coast, Kenya Medical Research Institute (KEMRI), Kilifi, Kenya; and School of Public Health, University of the Witwatersrand, South Africa; Institute for Human Development, Aga Khan University, Kenya; Institute for Human Development, Aga Khan University, Kenya; Centre for Geographic Medicine Research Coast, Kenya Medical Research Institute (KEMRI), Kilifi, Kenya; and Department of Psychiatry, University of Oxford, UK

**Keywords:** Problem Management Plus (PM+), mental health services, scoping review, common mental disorders, psychosocial interventions

## Abstract

**Background:**

Given the high rates of common mental disorders and limited resources, task-shifting psychosocial interventions are needed to provide adequate care. One such intervention developed by the World Health Organization is Problem Management Plus (PM+).

**Aims:**

This review maps the evidence regarding the extent of application and usefulness of the PM+ intervention, i.e. adaptability, feasibility, effectiveness and scalability, since it was introduced in 2016.

**Method:**

We conducted a scoping review of seven literature databases and grey literature from January 2015 to February 2024, to identify peer-reviewed and grey literature on PM+ around the world.

**Results:**

Out of 6739 potential records, 42 met the inclusion criteria. About 60% of the included studies were from low- and middle-income countries. Findings from pilot/feasibility trials demonstrated that PM+ is feasible, acceptable and safe. Results from definitive randomised controlled trials at short-term follow-up also suggested that PM+ is effective, with overall moderate-to-large effect sizes, in improving symptoms of common mental health problems. Although PM+ was more effective in reducing symptoms of common mental disorders, it was found to be costlier compared to usual care in the only study that evaluated its cost-effectiveness.

**Conclusions:**

Our findings indicate that PM+, in its individual and group formats, can be adapted and effectively delivered by trained helpers to target a wide range of common mental health concerns. More effectiveness and implementation evidence is required to understand the long-term impact of PM+, its cost-effectiveness and scalability, and moderators of treatment outcomes such as gender and delivery formats.

Mental health is a fundamental human right and a critical element in the sustainable development of societies worldwide, hence the need for increased investment in mental health services as part of universal health coverage.^1^ Nevertheless, mental health problems, particularly common mental disorders (CMDs) like depression, anxiety, stress and prolonged grief, remain among the top leading causes of the global burden of disease, with no evidence of a global reduction in the burden since 1990.^2^ Although mental illnesses occur across all levels of socioeconomic status, most populations in low-resource settings do not have adequate access to effective psychological and pharmacological interventions, with up to a 90% mental health treatment gap reported.^3^ Evidence-informed interventions such as cognitive–behavioural therapy have been found effective in some low- and middle-income countries (LMICs).^4,5^ Nonetheless, there are significant barriers to the sustainable delivery of such psychological interventions in LMICs, including limited mental health funding and infrastructure, inadequate psychological treatments adapted to the local context and challenges associated with their implementation, e.g. limited availability of mental health specialists.^6,7^

## An overview of the Problem Management Plus

Implementation of low-intensity psychological therapies by non-mental health specialists is one potential solution to these challenges, which is receiving significant attention globally.^8,9^ Problem Management Plus (PM+), developed by the World Health Organization (WHO), is a brief, low-intensity, five-session transdiagnostic psychological intervention that may be delivered by trained non-mental health specialists to address mental health treatment gaps in LMICs.^10^ The intervention can be delivered in individual or group format.^10,11^ Each PM+ session usually lasts 90 min. PM+ aims to alleviate symptoms of CMDs, including depression, anxiety and distress, regardless of whether exposure to adversity has caused these problems. The intervention also seeks to address self-identified practical problems (e.g. unemployment, interpersonal conflict) among adults. Over the five PM+ sessions, clients are taught four main strategies: (a) managing stress, (b) managing problems, (c) ‘get going keep doing’ and (d) strengthening social support. Within the first session, PM+ clients receive psychoeducation around common reactions to adversities. The last session focuses on relapse prevention. Although PM+ has often been delivered face to face, it has also recently been adapted to allow for remote delivery via videoconferencing tools,^12,13^ mobile telephones^14^ or online platforms.^15^

The intervention was initially developed for and successfully evaluated in LMICs, where mental health resources are often limited.^16–21^ Recently, it has also been investigated among refugees in high-income countries (HICs).^22–26^ Thus, the current study carries out a comprehensive scoping review to synthesise the evidence on PM+ intervention to date, regarding its adaptations (including local contextualisation process) and implementation (including testing for feasibility, acceptability, effectiveness and scalability) around the world. The results of this review will inform evidence-based decision-making regarding the adaptation and implementation of PM+, guide future research efforts and promote the delivery of cost-effective, high-quality mental health interventions for individuals and communities experiencing CMD symptoms in different parts of the world.

## Method

The methods for this scoping review were informed by the methodological framework outlined by Arksey and O'Malley^27^ and further advanced by Levac et al.^28^ We report our results based on the Preferred Reporting Items for Systematic Reviews and Meta-Analyses extension for scoping reviews (PRISMA-ScR).^29^

### Review aims and research questions

This scoping review aims to describe the evidence base for the PM+ intervention and distil key information pertaining to the targeted populations, adaptations made, format/mode of delivery, characteristics of PM+ helpers, acceptability, feasibility, effectiveness and scalability around the world. The overarching research question guiding the review was: What is the extent of application and usefulness of the PM+ intervention worldwide? Specific research questions included:

- Which settings/contexts/populations has the PM+ intervention been implemented in?
- What are the formats/modes of delivery of the PM+ intervention in these settings?
- What are the characteristics of the PM+ helpers, and what is the nature of their training and supervision in delivering the intervention?
- What are the common adaptations of the PM+ intervention from the original intervention?
- How feasible, acceptable, safe and scalable is the PM+ intervention?
- What is the clinical effectiveness and cost-effectiveness of the PM+ intervention?
- What are the barriers and facilitators to effectively implementing the PM+ intervention?

### Identification of relevant studies

We considered both peer-reviewed and grey literature for inclusion of articles to capture the full extent of application of the PM+ intervention across the world. Since the intervention was first introduced in 2016, we limited our search to start from January 2015 up to February 2024, and studies conducted in adult populations. There were no other applied limits (e.g. language restriction) during the search.

### Search strategy

We searched five electronic databases: PubMed, PsycINFO, Scopus, CINAHL and Web of Science. Additionally, we searched Cochrane and the WHO clinical trials registry platforms. We also searched the following grey literature sources: Open Gray, NGO search engine, the Mental Health Innovation Network database, the International Federation of the Red Cross and Red Crescent database, the Mental Health and Psychosocial Support Network database and the Scaling up psychological interventions with Syrian refugees (STRENGTHS) project database. The electronic literature search followed the three-step procedure proposed by the Joanna Briggs Institute.^30^ In the initial step, we conducted a preliminary search in PubMed. The search terms ‘problem management plus’ and ‘intervention’ were used. P.N.M., M.M. and A.K. analysed the keywords used in the titles and abstracts of the identified abstracts. In the subsequent step, relevant keywords were reviewed to compile a list of terms to guide the detailed literature search in all databases. The improved final search strategy was developed in consultation with A.A. and M.K.N., and applied to fit the specifications of each database. The search results from individual databases were retrieved and uploaded to Endnote library version X9 (Endnote Team, Clarivate Company, Philadelphia USA; see https://endnote.com/downloads/). Lastly, we also searched the references of included articles and reports to identify potentially relevant literature meeting our eligibility criteria. Details of the search strategy are highlighted in Supplementary File 1 available at https://doi.org/10.1192/bjo.2024.55.

### Study selection

Duplicate articles/reports were then removed before starting the two-stage selection process. Study titles and abstracts were reviewed for eligibility based on the inclusion and exclusion criteria highlighted below. The screening was done independently by two authors (P.N.M. and A.K.). The subsequent results were reviewed by the team. Full-text articles were retrieved and thereafter assessed for eligibility by P.N.M. and A.K.

### Inclusion criteria

The comprehensive inclusion criteria included the following:

- Study population: we considered a broad range of participants based on age. Studies were included if they reported outcomes for youth/young adults, adults or older adults.
- Outcome measures: articles were included if they reported findings on the PM+ intervention only, e.g. adaptation, acceptability, feasibility, clinical effectiveness and cost-effectiveness data.
- Geographic location: studies conducted anywhere in the world.
- Study design: any type of original empirical intervention research, including pilot and definitive randomised controlled trials (RCTs), quasi-experimental designs, pre–post evaluations, open trials, mixed method studies or other applicable study designs reporting on PM+, e.g. field experience reports on implementing PM+. Articles where PM+ was significantly adapted, thus deviating from the original PM+ strategies, were not considered PM+ and hence excluded.

### Exclusion criteria

Non-empirical studies, including scoping reviews, literature reviews, systematic reviews, meta-analyses and intervention protocols, were excluded. Studies reporting mental health interventions other than PM+ were also excluded.

### Data charting

The data extraction form was developed by P.N.M. in Microsoft Excel 365 (Microsoft Corporation, Redmond, USA; https://office.microsoft.com/excel), and reviewed by the study team. It was then piloted by P.N.M. and A.K., who independently extracted the data from a sample of 10% of the included studies. Differences were compared and discussed, and a final data extraction tool was developed. For the remaining articles, data extraction was independently conducted by P.N.M., M.M., M.K.N. and A.K. In cases of disagreements, the two reviewers re-evaluated the same article and reached a consensus. Categories included in the data extraction sheet included: (a) study and sample characteristics such as author, publication year, country of the study, study design and follow-up time (for RCTs), sample size, study population, inclusion and exclusion criteria, participants’ age and proportion of female participants; (b) intervention details such as group-based or individual, number of sessions delivered and duration for each, format/mode of delivery, PM+ helpers and their characteristics (e.g. education), PM+ training and supervision procedures, control details, study outcomes, the impact of intervention if evaluated, barriers and facilitators during the implementation of the intervention, and details of intervention adaptations.

### Collating, summarising and reporting of the results

We collated the findings based on the review questions to create a summary of the included studies by using a narrative synthesis approach. Articles were tabulated and grouped by study outcomes and study characteristics. Patterns were identified and translated to themes and further refined with an iterative process. The evidence was synthesised according to key review outcomes, i.e. PM+ adaptation, characteristics of PM+ helpers, PM+ feasibility, effectiveness, cost-effectiveness, and barriers and facilitators to PM+ implementation. Evidence was synthesised to provide a meaningful narrative relevant to the review questions.

## Results

### Search results

**Fig. 1:**
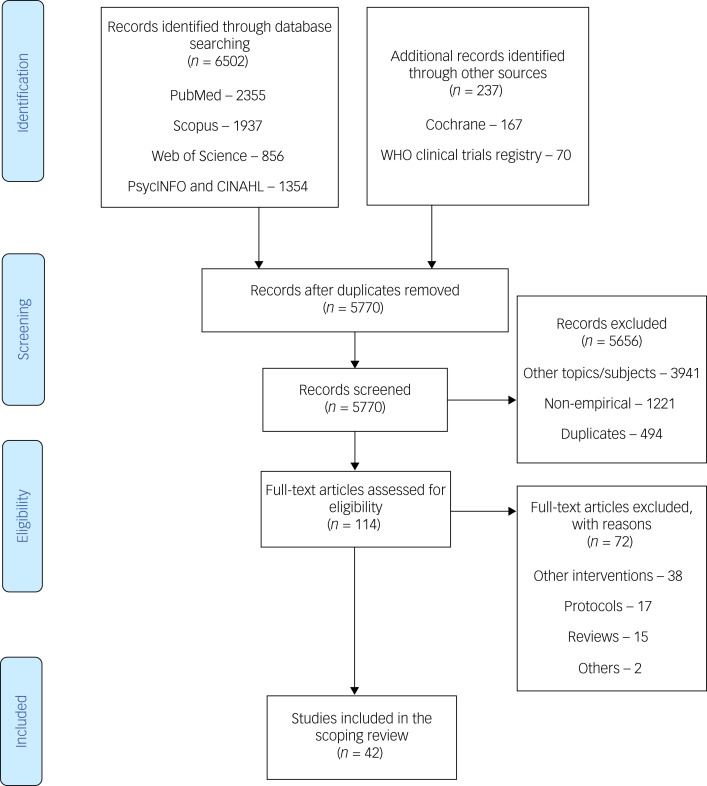
Preferred Reporting Items for Systematic Reviews and Meta-Analyses flow chart illustrating the study selection process. WHO, World Health Organization.

Figure 1 is the PRISMA flowchart highlighting the study identification and selection process. Our electronic database literature search identified 6502 records, and a further 237 were identified from the Cochrane database and WHO clinical trials registry. After excluding 969 duplicate records, we screened the titles and abstracts of 5770 records, of which 5656 were excluded. We then screened the full texts of the remaining 114 records, yielding 42 eligible articles. The reasons for exclusions are highlighted in Fig. 1.

### Characteristics of included studies

The characteristics of the 42 included studies published between 2016 and 2023 are presented in Supplementary File 2. The 42 studies originated from 19 countries across the world: Pakistan^17,20,31–33^ (*n* = 5), Jordan (*n* = 4),^18,34–36^ Kenya (*n* = 4),^14,16,37,38^ Nepal (*n* = 3),^19,39,40^ The Netherlands (*n* = 3),^23,24,41^ Turkey (*n* = 2),^42,43^ Austria (*n* = 2),^44,45^ China (*n* = 2),^46,47^ Switzerland (*n* = 2)^22,48^ Australia (*n* = 1),^12^ the UK (*n* = 1),^26^ Colombia (*n* = 1),^49^ Central African Republic (*n* = 1),^50^ Ethiopia (*n* = 1),^51^ the USA (*n* = 1),^52^ Spain (*n* = 1),^15^ Iraq (*n* = 1),^53^ Venezuela (*n* = 1)^49^ and Malaysia (*n* = 1).^54^ Five studies were conducted in multiple countries.^21,55–58^ Most of the included studies, about 60%, originated from LMICs.

Twenty-five (60%) of the studies were RCTs.^12,14–20,22–24,26,31–33,35,36,39,43,45–47,49,54,59^ Ten (40%) of these were feasibility or pilot RCTs documenting the feasibility, acceptability, safety and potential effectiveness of the PM+ intervention.^14,22,23,26,32,36,39,43,49,59^ The remaining 15 RCTs were definitive trials: 14 assessed clinical effectiveness and one evaluated cost-effectiveness. Apart from RCTs, two studies utilised a pre–post design.^50,53^ Eight studies used qualitative approaches to explore the cultural adaptation and/or report on feasibility, acceptability and scalability of the PM+ intervention.^34,37,40,41,44,48,52,58^ Five other studies were field case reports on the adaptation/implementation process and feasibility of the PM+ intervention.^21,51,55,57,60^ The remaining two studies used a theory of change (ToC) workshop to explore context-specific pathways of scaling up PM+, including the potential barriers and facilitators to scaling up the intervention.^42,56^

Supplementary File 3 highlights the primary and secondary outcomes targeted by experimental studies (*n* = 27). Twenty-five of the studies evaluated psychological distress as the main primary outcome, predominantly symptoms of depression and/or anxiety. Different measures of psychological distress were employed, including (a) the Hospital Anxiety and Depression Scale (HADS^61^), used in eight studies;^12,17,20,26,31–33,46^ (b) the Hopkins Symptom Checklist-25 (HSCL-25^62^), used in six studies;^18,23,24,35,36,43^ (c) the 12- and 28-item General Health Questionnaires,^63^ used in four studies;^16,19,45,59^ (d) the nine-item Patient Health Questionnaire (PHQ-9^64^), used in three studies;^14,39,53^ (e) the seven-item Generalised Anxiety Disorder Scale (GAD-7^65^), used in one study;^14^ (f) the Kessler Psychological Distress Scale (K10^66^), used in one study;^22^ (g) the Social Anxiety Scale for Children,^67^ used in one study;^47^ and (h) the Patient Health Questionnaire-Anxiety and Depression Scale (PHQ-ADS^68^), used in one study.^15^

In addition to psychological distress, three studies^22,33,53^ assessed daily functioning as a primary outcome, using the WHO Disability Assessment Schedule (WHODAS 2.0).^69^ Two studies^49,50^ assessed general well-being as a separate primary outcome, using the WHO Well-Being Index (WHO-5).^70^ Two other studies^49,53^ assessed participant self-identified problems as a primary outcome of interest, using the Psychological Outcomes Profiles Scale (PSYCHLOPS).^71^ Perera et al^49^ additionally included quality-of-life assessment with the WHO Quality of Life measure (WHOQOL-BREF).^72^

Three secondary outcomes were frequently assessed across studies implementing PM+. First, post-traumatic stress disorder (PTSD) symptoms were evaluated in 19 studies using various tools, including the International Trauma Questionnaire (ITQ) and PTSD Symptoms Checklist (PCL), which had variations such as PCL for DSM-5^73^ and PCL-C.^74^ Second, participant self-identified problems were assessed with the PSYCHLOPS in 15 studies. Lastly, in 14 studies, participants’ daily functioning was evaluated with the WHODAS 2.0. Nine studies^12,15,17,19,22,26,31-33^ that assessed psychological distress as a primary outcome concurrently evaluated mental health as a separate secondary outcome, mostly depressive disorder symptoms with the PHQ-9 (seven studies). Other secondary outcomes evaluated included perceived social support, self-reported health service utilisation, prolonged grief, prodromal psychotic symptoms, quality of life, prior exposure to stressful or traumatic life events, gender-based violence and post-migration stressors.

### Population targeted

Among the 27 experimental studies, 3879 clients from diverse populations and varying sample sizes ranging from 7 to 969, were recruited and received the PM+ intervention. Four of the 27 studies were exclusively conducted among women.^16,31,32,59^ Overall, 70% of the PM+ clients were women. All the experimental studies enrolled adults with moderate levels of common mental health problems, including psychological distress, anxiety, depression and impaired psychosocial functioning. Potential clients having significant cognitive or neurological impairment, acute medical conditions, severe mental disorders and imminent risk of suicide were excluded from the experimental studies. Various at-risk populations were targeted, including refugees/asylum seekers/internally displaced persons/migrants (*n* = 14),^18,22–24,26,31,35,36,43,45,49,50,53,54^ adult primary care attendees (*n* = 4),^17,20,32,33^ women with a history of gender-based violence (*n* = 2),^16,59^ adults from earthquake affected regions (*n* = 2),^19,39^ young adults living with HIV (*n* = 1),^14^ adults living with multiple myeloma (*n* = 1),^46^ adults affected by the COVID-19 pandemic (*n* = 1),^12^ parents of children living with autism spectrum disorder (*n* = 1)^47^ and healthcare workers working in COVID-19 pandemic hotspots (*n* = 1).^15^

### Format/mode of delivery of the intervention

Most of the included studies implemented all PM+ strategies. In studies where adaptations of PM+ was empirically evaluated (*n* = 26),^12,14–20,22–24,26,31–33,35,36,39,43,45–47,49,54,59^ it was mostly delivered to individual clients in 16 studies^14-17,20,22-24,33,45-47,49,53,54,59^ and with a group-based delivery approach in ten other studies.^12,18,19,31,32,35,36,39,43,50^ In the study by Dowrick et al,^26^ PM+ was delivered both individually and in groups. Group-based sessions comprised four (minimum) to ten (maximum) individuals.

The majority of the studies (*n* = 13) evaluated PM+ using its original format of five weekly face-to-face sessions, each lasting 90 min. Nine studies^18,19,31,32,35,36,39,45,50^ had similar formats, with the exception that either sessions were extended to between 2 and 3 h or sessions were done over 6 weeks.^45^ In some studies, PM+ was delivered online either through videoconferencing or live streaming, with session duration lasting 40 min^47^ or 60 min.^12,15^ de Graaf et al^24^ applied a hybrid approach of face to face and video calls, to deliver five weekly sessions of PM+, each for 90 min, whereas Nyongesa et al^14^ delivered PM+ strategies via mobile telephone calls for ten sessions, each lasting 45 min on average. In nine studies,^16,18,19,35,36,39,43,53,59^ PM+ helpers were gender-matched with PM+ clients. Supplementary File 4 provides further details.

### PM+ helpers and their training and supervision

Nearly all studies used PM+ helpers, largely from the community and with no prior training in mental health, for delivery of the PM+ strategies, except in three studies^12,33,45^ where mental health specialists (psychologists) were used as PM+ helpers. The non-specialist PM+ helpers included peer refugees or those with lived experience of the asylum process,^22–24,26,43^ lay healthcare workers or those in health-related disciplines, e.g. counsellors, nurses, social workers, community-based psychosocial workers, community health workers and Red Cross volunteers;^12,15–17,20,35,36,39,46,50,53,59^ PM+ helpers^14,31,32^ and local staff of the implementing non-governmental organisations.^19^ In 15 studies,^12,16–18,20,22–24,26,33,35,36,43,53,59^ the PM+ helpers underwent 8 days of training, usually from local trainers such as mental health specialists, mostly psychologists, trained as PM+ trainers or international master trainers from the WHO involved in the development of PM+. In the remaining studies, the longest training of the PM+ helpers was 20 days.^39^ Often, the training involved education about CMDs, basic helping skills, delivery of intervention strategies, facilitation/supervision skills, self-care and psychological first aid. The classroom training was subsequently followed by practice cycles of varied length under close supervision. In a few of the studies,^16,26,31,59^ the PM+ helpers had to complete competency assessments before offering PM+. Supervision was mostly cascaded from an international PM+ specialist to local mental health specialists and onward, to PM+ helpers. Most of the PM+ helpers received weekly face-to-face group supervision sessions (about 2 h) from local supervisors, who in turn received weekly to fortnightly supervision (1 to 2 h) from international trainers/supervisors, mostly via video conference software, e.g. Skype. Two studies did not report the details of PM+ helpers’ training/supervision.^46,47^

### PM+ adaptations reported

All of the 27 experimental studies included in the review reported adaptations of the PM+ intervention. The adaptation process varied among the studies, but generally included literature reviews, stakeholder engagements, qualitative explorations, literal translations, cognitive interviews and adaptation workshops. A few studies used established frameworks for their adaptation process, including the eight-element framework for the adaptation of psychological interventions^75^ and the ten-step mental health cultural adaptation and contextualisation for implementation (mhCACI) procedure.^40^ The first framework encompasses eight elements: language, therapeutic relationship, metaphors, intervention content, concept of illness, treatment goal, delivery methods and context. The mhCACI framework is a ten-step process: identifying mechanisms of action; conducting a literature desk review for the culture and context; conducting a training of trainers; translating intervention materials; conducting an expert read through all the materials; qualitative assessment of intervention population and site; conducting practice rounds; conducting an adaptation workshop with specialists and implementers; pilot testing the training, supervision and implementation; and reviewing through the process evaluation.

The core PM+ components and application/teaching of the main strategies were retained among the included studies. However, several changes were proposed across the intervention manual, training, supervision and implementation protocols. Generally, changes ranged from minor adjustments to terminology, broader changes and how metaphors, stories and illustrations were presented during the interventions. Some of the substantial adaptations included translation to local languages, addition or splitting of sessions, mode of delivery (e.g. face to face versus online, over the telephone or video conferencing) and session duration.^12,14,18–20,22–24,32,33,43,45,58,59^

### Feasibility, effectiveness and scalability of the PM+ intervention

#### Feasibility

Testing the feasibility of mental health interventions in local contexts is crucial in evidence translation.^76,77^ Several elements of intervention feasibility have been documented in the literature, including recruitment capability, data collection procedures, design procedures, retention of clients, optimal content and delivery, acceptability, adherence, the likelihood of cost-effectiveness, the capacity of providers to deliver the intervention and safety.^76–78^ In the current review, PM+ feasibility was reported in ten pilot RCTs,^14,22,23,26,32,36,39,43,49,59^ and qualitative and case studies.^21,37,44,51,52,55^ The frequently reported feasibility elements in these studies included recruitment and retention of PM+ clients, training and supervision of PM+ helpers, intervention fidelity, intervention safety, acceptability (by PM+ helpers and clients) and potential effectiveness of PM+ intervention. All except one of the pilot RCTs^26^ reported maximum recruitment of intended clients and reported high retention rates of their clients, usually more than 75% in most of the studies. Most of the studies utilised locally contextualised outcome measures and demonstrated the delivery of PM+ by trained helpers who were regularly supervised. PM+ was also found to be safe. Intervention safety was assessed by the extent of adverse events directly related to the intervention, e.g. a marked increase in suicidal thoughts or the presence of any serious adverse event. Some of the studies adopted a cut-off of ≥10% adverse events to delineate intervention safety. PM+ acceptability was largely assessed with qualitative studies where PM+ clients and helpers and other stakeholders (e.g. project staff and policy makers) reported a positive view of the intervention content, implementation and format. The only feasibility RCT with inconclusive findings had to be stopped because of prolonged governance issues following the onset of the COVID-19 pandemic; thus, the authors could not sufficiently provide clear conclusions regarding the feasibility of PM+ intervention.^26^ Although the feasibility trials were not statistically powered to detect statistically significant differences, significant differences in favour of PM+ clients were found for anxiety,^14,23,32^ depression^14,23,32^ and PTSD symptoms;^23^ functional impairment;^23,32^ self-identified problems^23^ and quality of life,^14^ suggesting the potential effectiveness of PM+ on these studied outcomes.

#### Effectiveness

The clinical effectiveness of PM+ was examined in 14 definitive RCTs^12,15–19,24,31,33,35,45–47^ and two pre–post trials^50,53^ (Supplementary File 5). Among studies evaluating effectiveness, all except one^26^ found a positive significant effect, with overall moderate-to-large effect sizes, in favour of PM+ for several primary outcomes, including depressive symptoms,^12,15,17,18,24,31,33,46,53^ anxiety symptoms,^12,15,17,24,31,33,46,47^ psychological distress,^16,19,45,46^ functional impairment^33,50,53^ and PTSD.^50^ All of these studies except one^35^ fixed their primary end points between 1 week and 6 months. Only one study evaluated the long-term effects of the PM+ intervention at 12 months’ follow-up, and the authors found no significant differences between treatment arms for depression and anxiety at this time point.^35^ Several studies also reported positive significant effects in favour of PM+ for a range of secondary outcomes, including self-identified problems,^16–18,24,31,45^ PTSD,^16,17,24,33,45^ depressive symptoms^15,17,19,24,33^ and anxiety symptoms.^15,24^ Improvements in functional impairment were also reported,^16,17^ as well as increases in positive parenting (e.g. a reduction in inconsistent disciplinary parenting that was associated with reductions in attentional and internalising problems in children),^18^ parenting stress,^47^ anhedonia, COVID-19-related fears and contamination^12^ and number of days off,^53^ and improvements in parent–child interaction,^47^social support,^47^ quality of life and emotion regulation,^45^ and ability to carry out usual activities.^53^

Among the frequently assessed outcomes (symptoms of anxiety, depression, post-traumatic stress, functional impairment and personally identified problems), there was no clear pattern of effect size superiority. Based on the delivery format, individual-based PM+ appeared to have superior effect sizes compared with group-based PM+ among primary outcomes. However, no clear patterns were observed based on remote versus face-to-face delivery formats. Virtually, all of the studies reporting effect sizes were delivered by lay helpers; hence, little comparison could be made based on this aspect. Relatedly, most of the PM+ clients in the review were women, and none of the studies evaluated gender-specific effects; hence, we cannot make gender-specific conclusions about effectiveness.

#### Cost-effectiveness

Cost-effectiveness of the PM+ intervention was examined in only one study.^20^ The cost of delivering PM+ per participant was estimated at US$163 (using an international trainer and supervisor) and US$35 (employing a local trainer). In addition, the mean cost per unit score improvement in anxiety and depressive symptoms on the HADS was US$28 with an international trainer/supervisor and US$6 with a local trainer/supervisor. The mean incremental cost-effectiveness ratio to successfully treat a case of depression (PHQ-9 score ≥10) was US$517 with an international supervisor compared with US$103 with a local supervisor. Additional cost-effectiveness data from different organisations, including the STRENGTHS Consortium, is expected in the coming months.^79^

#### Scalability

None of the included studies described the scale-up of the PM+ intervention empirically. However, scalability potential was examined by five studies, three qualitative studies^34,41,48^ and two ToC workshop reports.^42,56^ The qualitative studies sought to identify the factors that may influence the process of the large-scale implementation of PM+ and to develop recommendations to guide the implementation process. In one of these studies,^48^ the authors documented three significant themes likely to affect the longer-term implementation of PM+: preconditions for successful integration in the health system before scaling up, e.g. sustainable funding and introducing a stepped care approach; PM+ requirements to support scale-up (e.g. quality control during its delivery, modality, time and setting being offered) and the perceived benefits of scaling up. Similarly, the second qualitative study^34^ recommended sustainable funding to be made available for staff, training, supervision, infrastructure, coordination, expansion and evaluation of the scaling up of the PM+ intervention. The findings of the last qualitative study^41^ suggested that the wider implementation of PM+ would largely depend on addressing essential barriers such as stigma, attrition, fragmentation, legal and financial challenges. Also noteworthy was the formalisation of non-specialist PM+ helpers, with accompanying structures for accreditation and supervision.^41^

The two ToC workshop articles documented the practical steps of scaling up the PM+ intervention to help understand the change process of the intervention and map out causal pathways through which it has an effect. The results highlight that the scaling up of PM+ requires careful planning and investment from different stakeholders at the national level. Two distinct causal pathways for scale-up were reported: a policy and financing pathway and a health services pathway, which are interdependent.

### Barriers and facilitators to effective implementation of the PM+ intervention

The 12-month follow-up duration of the intervention posed a limitation, lacking long-term benefits and sometimes worsening symptoms of mental health issues.^35^ The COVID-19 pandemic introduced additional challenges, causing delays in implementation because of governance issues and supply shortages.^26^ Structural barriers included PM+ clients’ engagement in income-generating activities or household chores, leading to session delays and limited attendance.^32,36^ Attitudinal barriers driven by mismatched expectations complicated implementation, influenced by a prior non-governmental organisation.^32,37,39^ High implementation costs,^20,24,59^ security threats^17^ and transportation issues further hindered progress. Confidentiality within group interventions affected participation,^32^ whereas challenges accessing referral services limited additional mental health treatment.^39^ Complexity of strategies^49^ and psychological barriers posed challenges, along with general job dissatisfaction among PM+ helpers. Sociocultural barriers, such as cultural explanations of mental health issues, were identified as important barriers in one of the studies.^50^ Low mental health literacy and lack of trust contributed to high attrition in PM+ implementation in another study.^45^

Competent PM+ helpers, supported by comprehensive training, live supervision, self-care strategies and organisational commitment, ensured safe and effective intervention delivery.^19,39,49–51^ Cultural adaptation, that is, adapting the intervention to specific contexts, was essential.^14,18,19,22,23,32,33,43,45,58,59^ Gender-matched assessors alleviated fears in settings where perceptions mattered.^31,39^ Early stakeholder engagement, partnering with non-governmental organisations and local government, maximised resources and accessibility.^43,49^ PM+ programmes delivered by culturally sensitive non-mental health specialists provided scalable solutions,^24,33,58^ supported by task-shifting approaches.^17,20,59^ Community engagement through sensitisation events built trust,^49^ and the group format fostered acceptance and motivation, especially among women.^37,39^ Amid the pandemic, videoconferencing facilitated PM+ delivery and addressed mental health issues.^12^

## Discussion

The current review provides a reflection on the current state of the literature on PM+ intervention regarding its application and usefulness worldwide. We identified 42 studies, including 25 RCTs and two pre–post studies, with 3879 PM+ clients, most of whom were women. Our results show that PM+ has been adapted and implemented in various settings and populations in both HICs and LMICs, with most evidence, about 60% of included studies, coming from LMICs. The PM+ intervention underwent adaptation in all the included experimental studies, which essentially guided how it was delivered, e.g. group-based versus individual formats; face to face versus online, over the telephone or videoconferencing; number and duration of sessions. The vast majority of PM+ helpers, almost exclusively, are non-mental health specialists, largely from the community and with no prior training in mental health. Findings from the pilot/feasibility RCTs generally indicated that PM+ in group or individual formats is a feasible, acceptable and safe psychological intervention. Results from definitive RCTs at short-term follow-up also suggest that PM+ is efficacious, with overall moderate-to-large effect sizes, in improving symptoms of depression, anxiety, psychological distress, PTSD and functional impairment. Although PM+ was more effective in reducing symptoms of CMDs, it was found to be costlier compared with usual care alone in the only study that evaluated its cost-effectiveness at 3 months. None of the included studies described the scaling up of the PM+ intervention; however, potential scalability was examined, identifying the factors/pathways of scaling up the intervention.

Our findings on PM+ effectiveness are consistent with those of a recent global meta-analysis of scalable psychological interventions including PM+,^80^ confirming that PM+ is effective in reducing distress and promoting positive mental health in people exposed to adversities. Nonetheless, there is still a need for larger high-quality evidence including research on participant-level moderators of the effects of these interventions, and studies that evaluate cost-effectiveness and how to best integrate PM+ into stepped care programmes.^80^ Efforts are already underway, and such research is being prepared.^81–86^

Our observation that most of the existing research on PM+ is concentrated in LMICs is expected. The mental health treatment gap is often more pronounced in these areas; therefore, the case for testing scalable psychological interventions would be significantly stronger.^3^ This is very encouraging and aligns with the WHO's Mental Health Gap Action Programme (mhGAP),^87^ which aims to scale-up services for mental, neurological and substance use disorders for countries, especially within LMICs.^88,89^ Our results also add to the growing interest in low-intensity interventions in a few HICs as part of a stepped care model to address the mental health needs of communities affected by adversity as they use fewer resources, making them more scalable. A growing body of evidence indicates that the adapted forms of therapy, such as cognitive–behavioural therapy and interpersonal therapy, can be effective in different cultures around the world when they are culturally adapted. Our review found that PM+ research was frequently conducted in populations affected by humanitarian crises, including refugees/asylum seekers/internally displaced, residents of earthquake-prone areas and COVID-19 pandemic hotspots, in about two-thirds of the experimental studies. Other populations included women with a history of gender-based violence, emerging adults living with HIV, adults in primary care settings, adults living with multiple myeloma and parents of children living with autism spectrum disorder. As it is established in the literature,^90–93^ people in the abovementioned population groups have an increased risk of developing CMDs and could thus benefit from PM+.

Noteworthily, almost three-quarters of PM+ clients in the included experimental studies were women. It is possible that existing mental health treatments are not engaging or accessible to men.^94,95^ Past research has shown that men are less likely than women to seek help for mental health problems, including CMDs.^96,97^ This difference could be a result of stigma associated with mental illness or cultural attitudes, e.g. where men are ‘allowed’ to express their distress through alcohol and substance misuse, whereas it is more ‘accepted’ for women to express their distress.^98^ Some men view mental health problems and help-seeking as a sign of weakness.^99^ Frequently reported barriers to male participation include stigma of mental illness, illness severity, confidentiality concerns, inconvenience, transport challenges and lack of financial reward.^100^ The underrepresentation of men in PM+ is significant. More research should be conducted to understand why this is the case, and encourage approaches that are engaging and appealing for men to enhance access.

Cultural adaptation of content and implementation strategies are needed for effective scale-up of psychological interventions, and different frameworks exist to guide this process.^40,75,101,102^ Meta-analytic evidence indicates that culturally adapted psychological interventions are effective when compared with a variety of control conditions,^103^ and more effective than unadapted versions of the same interventions in direct comparison.^104^ In our review, all of the experimental studies reported PM+ adaptation using different methods or frameworks. This led to changes in the intervention manual, training, supervision and implementation/delivery protocols. Expectedly, all except one of the pilot/feasibility trials found PM+, both group and individual formats, feasible, acceptable and safe, evidenced by high retention rates and implementation by trained and supervised non-specialist PM+ helpers.

The intersection of technology and mental health services has brought revolutionary changes in confronting mental health challenges for the past two decades. Digital technologies have enhanced mental health services by offering effective and timely solutions that scale-up and decentralise mental healthcare across several platforms, e.g. mobile health applications, teletherapy and web-based interventions.^105,106^ These advancements provide innovative solutions and make mental health services accessible and affordable. The COVID-19 pandemic placed a tremendous strain on research activities, e.g. the halting of research studies, as shown in one of the studies included in this review.^26^However, it also created a unique opportunity for greater application of these technologies in mitigating the pandemic-driven spike in mental health problems.^105,107,108^ In our review, five studies leveraged technology to implement PM+ through videoconferencing or live streaming,^12,15,47^ mobile telephone calls^14^ and video calls.^24^ Four of these studies^12,14,15,24^ were implemented during the COVID-19 pandemic period. Findings from some of these studies^12,14^ provide initial evidence of leveraging technology in implementing PM+ in addition to the face-to-face delivery formats, and may offer viable and scalable approaches to mitigating mental health problems during crises. Further research is needed to provide a nuanced understanding on the application of different digital technologies in the implementation of PM+.

Our review also found modest benefits of PM+ in the short term compared with control clients in improving symptoms of depression, anxiety and PTSD, psychological distress, and functional impairment, where all of these were studied as primary outcomes. These findings suggest that PM+ is efficacious, at least in the short term, and adds to the growing body of research on the effectiveness of brief, low-intensity psychological interventions that may be used to reduce the prevailing mental health treatment gap. Our finding on the effectiveness of PM+ in this review is limited only in the short term. Only one RCT evaluated the long-term impact of PM+ at 12 months;^35^ however, additional data is expected from the STRENGTHS Consortium.^79^ In our review, PM+ did not show any benefit at the primary end point of 12 months, in contrast to the observation that at 3 months, the intervention led to higher reductions in depressive symptoms. This finding is consistent with previous evidence reporting that the benefits of psychological interventions do not consistently persist despite initial improvement in symptoms.^109,110^ Given the limited research in this area, the findings point to the need for more evaluation of the long-term impact of PM+. As it stands, however, the challenge is in developing ongoing programmes that can sustain the initial gains of effective interventions. One way to address this issue is by delivering booster sessions or practice reminders for the PM+ strategies. The use of stepped care programmes might further triage people with severe distress to more intensive programmes, whereas those with moderate distress may still benefit from brief interventions. Booster sessions have been shown to be effective in preventing relapse for a range of mental health conditions, including CMDs.^111,112^ Booster sessions can also benefit clients who did not attend all the intervention sessions, giving them a chance to learn or reinforce these strategies. We also cannot make gender-specific conclusions about the effectiveness of PM+, as this was not reported in the included studies. Future studies could further consider a gender perspective through research and implementation practices.

PM+ was also associated with significant positive improvements in a range of secondary outcomes, including personally identified problems, quality of life, social support, COVID-19-related fears and contamination, parenting stress and reductions in inconsistent disciplinary parenting that resulted in reductions in attentional and internalising problems in children. These findings suggest that PM+ is positively associated with broader and multidimensional aspects across the health spectrum.

Significant progress has been made in terms of the availability of evidence-based mental health packages for populations affected by adversity, especially in LMICs. Nonetheless, the scalability and sustainability of such mental health interventions remain an important challenge in LMICs. None of the included studies described the scale-up of the PM+ intervention; however, a few studies examined the potential scale-up by describing the pathways and factors influencing scale-up. For PM+ to be successfully scaled up, it is critical for it to be effectively integrated into existing health systems, as well as assessing the perceived benefit among potential recipients and addressing any demand gaps. Additionally, factors that are likely to enhance the scale-up, such as quality and delivery methods, need to be examined and improved. Also important is addressing prevailing barriers, e.g. mental health stigma. Although none of the research papers described/examined the actual scale-up of PM+, we found several reports from grey literature search that reported on the routine implementation of PM+ in different settings. The Kenyan Ministry of Health created a service delivery framework for the implementation of PM+, outlining how the national and county Ministries of Health link together and support county-level centres of excellence for long-term sustainable training and support of PM+ helpers, who are then linked to primary and community health units.^113^ Between 2013 and 2018, 1697 PM+ helpers and 20 master trainers had been trained. Additionally, 70 representatives from eight community-based organisations have also been trained in leadership, governance and business planning to ensure sustainability. In 2021, a total of 608 people (social workers, refugee volunteers and psychologists) were also trained on PM+ in Cameroon, Egypt, Ethiopia, Greece, Kenya, Niger, Peru and Sudan.^114,115^ Another 351 PM+ helpers and supervisors have also been trained in Rwanda, Peru, Mexico and Malawi.^57^ Over 100 people have also been trained on group PM+ to facilitate national coverage of PM+ for refugees across Jordan.^116^ Additional data is expected for Venezuelan women refugees and migrants in Colombia.^117^ From these reports, ownership of the programme by the local government, infrastructure investments and relevant training and supervision for PM+ helpers are critical for the transition to scale. More research is needed to assess the barriers and facilitators to routine PM+ implementation to inform further application.

One of the studies^20^ evaluated the cost-effectiveness of the PM+ intervention compared with enhanced usual care for CMDs in primary healthcare in Pakistan, to inform its potential scale-up. PM+ was found to be more effective, but also more costly, than enhanced usual care in reducing symptoms of depression and anxiety and improving functioning. This finding is consistent with evidence from LMICs on the cost-effectiveness of a task-shifting approach of delivering mental health interventions for treating CMDs delivered by primary care physicians.^118,119^ With the current model of training and supervision by international trainers/supervisors, PM+ was five times more costly for treating a person with depression compared with the cost of training and supervision by local trainers. This emphasises the importance of building up local capacity, working with local communities and mobilising local resources, as recommended by the Inter-Agency Standing Committee guidelines in mental health and psychosocial support (MHPSS) interventions.^120^ Additional cost-effectiveness is soon expected from the STRENGTHS Consortium.^121,122^ Preliminary evidence shows that at 3 months, PM+ is likely to have better outcomes, but at higher costs than usual care. The upcoming 12-month analysis will shed more light on this.

Task-shifting is a promising approach to addressing healthcare shortages, especially in LMICs. Accumulating evidence, especially from LMICs, shows that task-shifting can be a cost-effective approach to enhancing access to healthcare and improving patient outcomes for different health problems.^123–126^ However, there are few published reports on the cost-effectiveness of task-shifting psychological interventions in global mental health.^118,119,127–129^ Our finding that PM+ was more expensive compared with usual care alone in the only study that assessed its cost-effectiveness is consistent with previous studies involving other mental health interventions.^127,128^ Given the limited number of studies and mixed findings, more studies are needed to shed more light on this subject. Without appropriate design and planning, e.g. adapting models into local contexts and enabling policy environment, task-shifting may increase system costs or minimise efficiency, and not be as effective as envisaged.^124^ In a review of reviews, Heller et al identified various health system barriers and facilitators of task-shifting regarding each of the WHO's health system building blocks.^130^ Findings consistently identified six important lessons for successful task-shifting, including careful staff recruitment, comprehensive training, authorisation to provide autonomous care, sufficient medications and supplies, reliable data systems and fair performance-based compensation.

### Limitations

We did not appraise the quality of the included studies; however, this is a primary limitation of scoping reviews. Relatedly, we also did not pre-register the scoping review protocol. We also did not conduct a local consultation in regard to the Arksey and O'Malley scoping review framework, because of limited resources.

### Future research

Most of the included RCTs measured effectiveness in the short term; thus, the long-term effects of PM+ are largely unknown. We recommend that future studies explore this area. Similarly, additional studies on the cost-effectiveness and scalability of the PM+ are needed. The majority of the PM+ clients in the review were women. Future studies need to assess the effectiveness of PM+ specifically for men, or include large enough sample sizes of men and provide gender-specific results. We were also unable to map specific roles, training infrastructure, supervision structures and remuneration models for the non-specialist PM+ providers, pointing to potential directions for future policy and research initiatives. It is not evident within the research who is making decisions about their qualifications outside the respective study authors and investigators.

In conclusion, since its introduction in 2016, the PM+ intervention has been adapted and implemented in various vulnerable populations, primarily in humanitarian populations in HICs and LMICs. Our scoping review has summarised PM+ evidence to date regarding its adaptation, feasibility, effectiveness and potential scale-up, subsequently identifying important implications for policy makers, practitioners and local communities. Our review found a large variation in PM+ implementation, e.g. in terms of delivery methods, training and supervision of PM+ helpers. Potential implementors of PM+ need to be aware of these issues as they select, adapt and implement the variants of PM+ to maximise positive intervention outcomes and sustainability. Our findings also emphasise the crucial need to build local capacity, which may further drive scale-up. There is also scope for a common framework for training, supervising and implementing PM+ among stakeholders.

## Supporting information

Mwangala et al. supplementary material 1 Mwangala et al. supplementary material

Mwangala et al. supplementary material 2 Mwangala et al. supplementary material

Mwangala et al. supplementary material 3 Mwangala et al. supplementary material

Mwangala et al. supplementary material 4 Mwangala et al. supplementary material

Mwangala et al. supplementary material 5 Mwangala et al. supplementary material

## Data Availability

Data availability is not applicable to this article as no new data were created or analysed in this study. Additional data/information has been shared in the Supplementary Material.
