# Supplementary material for: A scoping review of the literature on the application and usefulness of the Problem Management Plus (PM+) intervention around the world
Source: BJPsych Open. 2024 Apr 23;10(3):e91. doi: 10.1192/bjo.2024.55 (PMC11060090; doi:10.1192/bjo.2024.55)
Supplement: Mwangala et al. supplementary material 1 — Mwangala et al. supplementary material [file S2056472424000553sup001.docx]

**Supplementary file 1|Search strategy**

We adopted a stepwise approach that involves first combining shorter keywords for each part of the PICOS criteria with “OR” and later checking the history for the list of searches and combining them with “AND”. We limited our search to studies conducted from Jan 2015 to Feb 2024.

The following search terms were used:

- ‘Problem management plus’ OR ‘group problem management plus’ OR ‘individual problem management plus’ OR pm+ OR group pm+
- Intervention OR program OR programme OR adaptation OR implementation OR scale up
- ‘world health organization’ OR who
- young adults OR adults

**PubMed search – 05/02/2024**

(((("problem"[All Fields] OR "problem s"[All Fields] OR "problems"[All Fields]) AND ("manage"[All Fields] OR "managed"[All Fields] OR "management s"[All Fields] OR "managements"[All Fields] OR "manager"[All Fields] OR "manager s"[All Fields] OR "managers"[All Fields] OR "manages"[All Fields] OR "managing"[All Fields] OR "managment"[All Fields] OR "organization and administration"[MeSH Terms] OR ("organization"[All Fields] AND "administration"[All Fields]) OR "organization and administration"[All Fields] OR "management"[All Fields] OR "disease management"[MeSH Terms] OR ("disease"[All Fields] AND "management"[All Fields]) OR "disease management"[All Fields]) AND "plus"[All Fields]) OR (("group s"[All Fields] OR "grouped"[All Fields] OR "grouping"[All Fields] OR "groupings"[All Fields] OR "groups s"[All Fields] OR "population groups"[MeSH Terms] OR ("population"[All Fields] AND "groups"[All Fields]) OR "population groups"[All Fields] OR "group"[All Fields] OR "social group"[MeSH Terms] OR ("social"[All Fields] AND "group"[All Fields]) OR "social group"[All Fields] OR "groups"[All Fields]) AND ("problem"[All Fields] OR "problem s"[All Fields] OR "problems"[All Fields]) AND ("manage"[All Fields] OR "managed"[All Fields] OR "management s"[All Fields] OR "managements"[All Fields] OR "manager"[All Fields] OR "manager s"[All Fields] OR "managers"[All Fields] OR "manages"[All Fields] OR "managing"[All Fields] OR "managment"[All Fields] OR "organization and administration"[MeSH Terms] OR ("organization"[All Fields] AND "administration"[All Fields]) OR "organization and administration"[All Fields] OR "management"[All Fields] OR "disease management"[MeSH Terms] OR ("disease"[All Fields] AND "management"[All Fields]) OR "disease management"[All Fields]) AND "plus"[All Fields]) OR (("individual s"[All Fields] OR "individualisation"[All Fields] OR "individualise"[All Fields] OR "individualised"[All Fields] OR "individualising"[All Fields] OR "individualism"[All Fields] OR "individualisms"[All Fields] OR "individualities"[All Fields] OR "individuality"[MeSH Terms] OR "individuality"[All Fields] OR "individualization"[All Fields] OR "individualize"[All Fields] OR "individualized"[All Fields] OR "individualizes"[All Fields] OR "individualizing"[All Fields] OR "individually"[All Fields] OR "individuals"[All Fields] OR "individuate"[All Fields] OR "individuated"[All Fields] OR "individuates"[All Fields] OR "individuating"[All Fields] OR "individuation"[MeSH Terms] OR "individuation"[All Fields] OR "individuations"[All Fields] OR "persons"[MeSH Terms] OR "persons"[All Fields] OR "individual"[All Fields]) AND ("problem"[All Fields] OR "problem s"[All Fields] OR "problems"[All Fields]) AND ("manage"[All Fields] OR "managed"[All Fields] OR "management s"[All Fields] OR "managements"[All Fields] OR "manager"[All Fields] OR "manager s"[All Fields] OR "managers"[All Fields] OR "manages"[All Fields] OR "managing"[All Fields] OR "managment"[All Fields] OR "organization and administration"[MeSH Terms] OR ("organization"[All Fields] AND "administration"[All Fields]) OR "organization and administration"[All Fields] OR "management"[All Fields] OR "disease management"[MeSH Terms] OR ("disease"[All Fields] AND "management"[All Fields]) OR "disease management"[All Fields]) AND "plus"[All Fields]) OR ("precis med"[Journal] OR "phys med"[Journal] OR "pharmacogn mag"[Journal] OR "pediatr med"[Journal] OR "pm"[All Fields]) OR (("group s"[All Fields] OR "grouped"[All Fields] OR "grouping"[All Fields] OR "groupings"[All Fields] OR "groups s"[All Fields] OR "population groups"[MeSH Terms] OR ("population"[All Fields] AND "groups"[All Fields]) OR "population groups"[All Fields] OR "group"[All Fields] OR "social group"[MeSH Terms] OR ("social"[All Fields] AND "group"[All Fields]) OR "social group"[All Fields] OR "groups"[All Fields]) AND ("precis med"[Journal] OR "phys med"[Journal] OR "pharmacogn mag"[Journal] OR "pediatr med"[Journal] OR "pm"[All Fields]))) AND ("intervention s"[All Fields] OR "interventions"[All Fields] OR "interventive"[All Fields] OR "methods"[MeSH Terms] OR "methods"[All Fields] OR "intervention"[All Fields] OR "interventional"[All Fields] OR ("program"[All Fields] OR "program s"[All Fields] OR "programe"[All Fields] OR "programed"[All Fields] OR "programes"[All Fields] OR "programing"[All Fields] OR "programmability"[All Fields] OR "programmable"[All Fields] OR "programmably"[All Fields] OR "programme"[All Fields] OR "programme s"[All Fields] OR "programmed"[All Fields] OR "programmer"[All Fields] OR "programmer s"[All Fields] OR "programmers"[All Fields] OR "programmes"[All Fields] OR "programming"[All Fields] OR "programmings"[All Fields] OR "programs"[All Fields]) OR ("program"[All Fields] OR "program s"[All Fields] OR "programe"[All Fields] OR "programed"[All Fields] OR "programes"[All Fields] OR "programing"[All Fields] OR "programmability"[All Fields] OR "programmable"[All Fields] OR "programmably"[All Fields] OR "programme"[All Fields] OR "programme s"[All Fields] OR "programmed"[All Fields] OR "programmer"[All Fields] OR "programmer s"[All Fields] OR "programmers"[All Fields] OR "programmes"[All Fields] OR "programming"[All Fields] OR "programmings"[All Fields] OR "programs"[All Fields]) OR ("acclimatization"[MeSH Terms] OR "acclimatization"[All Fields] OR "adaptation"[All Fields] OR "adaptations"[All Fields] OR "adapt"[All Fields] OR "adaptabilities"[All Fields] OR "adaptability"[All Fields] OR "adaptable"[All Fields] OR "adaptational"[All Fields] OR "adaptative"[All Fields] OR "adapte"[All Fields] OR "adapted"[All Fields] OR "adapting"[All Fields] OR "adaption"[All Fields] OR "adaptions"[All Fields] OR "adaptive"[All Fields] OR "adaptively"[All Fields] OR "adaptiveness"[All Fields] OR "adaptivity"[All Fields] OR "adapts"[All Fields]) OR ("implementability"[All Fields] OR "implementable"[All Fields] OR "implementation"[All Fields] OR "implementation s"[All Fields] OR "implementational"[All Fields] OR "implementations"[All Fields] OR "implementer"[All Fields] OR "implementers"[All Fields] OR "implemention"[All Fields]) OR (("scale s"[All Fields] OR "scaled"[All Fields] OR "scaling"[All Fields] OR "scalings"[All Fields] OR "weights and measures"[MeSH Terms] OR ("weights"[All Fields] AND "measures"[All Fields]) OR "weights and measures"[All Fields] OR "scale"[All Fields] OR "scales"[All Fields]) AND "up"[All Fields])) AND ("world health organisation"[All Fields] OR "world health organization"[MeSH Terms] OR ("world"[All Fields] AND "health"[All Fields] AND "organization"[All Fields]) OR "world health organization"[All Fields] OR "who"[All Fields]) AND ("young adult"[MeSH Terms] OR ("young"[All Fields] AND "adult"[All Fields]) OR "young adult"[All Fields] OR ("young"[All Fields] AND "adults"[All Fields]) OR "young adults"[All Fields] OR ("adult"[MeSH Terms] OR "adult"[All Fields] OR "adults"[All Fields] OR "adult s"[All Fields]))) AND (2015:2023[pdat])

Number of hits = 2,761
