# Supplementary material for: A scoping review of the literature on the application and usefulness of the Problem Management Plus (PM+) intervention around the world
Source: BJPsych Open. 2024 Apr 23;10(3):e91. doi: 10.1192/bjo.2024.55 (PMC11060090; doi:10.1192/bjo.2024.55)
Supplement: Mwangala et al. supplementary material 2 — Mwangala et al. supplementary material [file S2056472424000553sup002.docx]

**Supplementary file 2. Characteristics of included studies**

| **Author, year & country** | **Population; sample size; % female; age ±SD** | **Study design/**  **methods** | **Inclusion criteria** | **Exclusion criteria** | **Primary outcomes & follow-up time** | **Secondary outcomes** | **Control type** | **Intervention description** | **Intervention training & delivery** | **Supervision & quality control** | **Findings** |
| --- | --- | --- | --- | --- | --- | --- | --- | --- | --- | --- | --- |
| Acarturk, C., et al (2022); Turkey | Syrian refugees with psychological distress.  46 (24 in treatment arm); 67.4% female; 38.0 ±10.9 | two arm pilot RCT; 3-month follow-up. | psychological distress (K10>15); impaired psycho‑social functioning (WHODAS >16); ≥18 years; being Syrian; Arabic speakers | acute medical condition; suicide risk; severe mental disorder; severe cognitive impairment | symptoms of depression and anxiety at 3-month | psychosocial functioning; PTSD symptoms; self-identified problems | enhanced care as usual: free access to health services, leaflet with information on available community mental services | Group PM+ delivered face-to-face by lay facilitators. Participants received 5 sessions of gPM+ & ECAU delivered over 5 weeks. | Peer refugees (≥12 years of education) went through 8-day training from PM+ trainers. PM+ groups had 8-10 people. Facilitators matched by gender to the PM+ groups | Weekly group supervision from PM+ trainers. 10% of the sessions selected for fidelity assessment using a checklist. | 75% retention. Study not powered to show treatment effect. No significant differences in the primary and secondary outcomes between gPM+ and the ECAU. gPM+ was safe, acceptable, and feasible. |
| Akhtar, Aemal, et al (2021); Jordan | Syrian refugees with psychological distress.  64 (35 in treatment arm); 70% women; 43.0±7.2 | two-arm feasibility RCT | Syrian adults aged ≥18 years; parent of a child aged 10– 16 years; experiencing psychological distress (K10 ≥16) & disability (≥17 on WHODAS) | developmental, cognitive, or neurological impairments; severe mental disorder & imminent risk of suicide | Psychological distress at 1-week post-assessment | Symptoms of PTSD, grief, prodromal psychosis, and psychological distress in children of participants | Enhanced treatment as usual: home visit and information on where to seek mental health services and other helpful information e.g. parenting. Sessions took 15 minutes | Group PM+ delivered face-to-face by lay facilitators. Participants received 5 sessions of gPM+ & ECAU delivered over 5 weeks. Each session took 120 minutes | Lay helpers (BSc psychology or related field & Arab speaking) trained for 8 days. Helpers completed 2 practice cycles. Each session delivered by 2 helpers in gendered groups of 6-12 adults. | One local supervisor working within the camp provided weekly supervision. | Retention in gPM+ was (86%). Study not powered to show treatment effect. Safe and acceptable. Children whose parents got gPM+ had greater reductions in internalizing and externalizing symptoms. |
| Bryant, Richard A., et al (2022); Jordan | Syrian refugees aged ≥18 years screening positive for distress and impaired functioning  410 (204 in treatment arm); 73.2% women; 40.0±7.0 | single-blind, parallel RCT | ⩾18 years; psychological distress  (K10 ≥16); Arabic-speaking; disability (≥17 on WHODAS);  with a child/ dependent living in the household aged 10 to 16 years | significant cognitive or neurological impairment; acute medical conditions; severe mental disorders; and acute risk of suicide | Symptoms of depression & anxiety assessed at baseline, 6 weeks, and 3 months | disability; posttraumatic stress; personally identified problems; grief, prodromal psychotic symptoms, parenting behavior, and children’s mental health | Enhanced usual care: involving referral information for psychosocial services | Group PM+ delivered face-to-face by lay facilitators. Participants received 5 sessions of gPM+ delivered over 5 weeks. Each session took 120 minutes | Lay helpers (BSc psychology or related field) trained for 8 days. Helpers completed 2 practice cycles. Delivered by 2 helpers in gendered groups of 8-10 adults. | Weekly supervision from a local supervisor who received fortnightly supervision from a PM+ trainer. 20% of sessions attended by supervisor for treatment fidelity. | Retention – 82.4% in gPM+ and 91.7% in EUC. gPM+ showed greater reductions of depression, personally identified problems and inconsistent disciplinary parenting than EUC. |
| **Author, year & country** | **Population; sample size; % female; age ±SD** | **Study design/**  **methods** | **Inclusion criteria** | **Exclusion criteria** | **Primary outcomes & follow-up time** | **Secondary outcomes** | **Control type** | **Intervention description** | **Intervention training & delivery** | **Supervision & quality control** | **Findings** |
| Bryant, Richard A., et al (2022); Jordan | Syrian refugees aged ≥18 years screening positive for distress and impaired functioning  410 (204 in treatment arm); 70.2% women; 40.3±7.0 | Single-blind, parallel RCT | ⩾18 years; psychological distress  (K10 ≥16); Arabic-speaking; disability (≥17 on WHODAS);  with a child/ dependent living in the household aged 10 to 16 years. | significant cognitive or neurological impairment; acute medical conditions; severe mental disorders; and acute risk of suicide | Symptoms of depression & anxiety assessed at baseline, 6 weeks, and 3 months and 12 months. | disability; posttraumatic stress; personally identified problems; grief, prodromal psychotic symptoms, parenting behavior, and children’s mental health | Enhanced usual care: 15-minute visit to client’s caravan to share referral information for psychosocial services e.g., mental health and vocational training | Group PM+ delivered face-to-face by lay facilitators. Participants received 5 sessions of gPM+ & ECAU in 5 consecutive weeks. Each session took 120 minutes | Lay helpers (BSc psychology or related field) trained for 8 days and thereafter, completed 2 gPM+ practice cycles. Each gPM+ had 6-12 clients and was led by 2 helpers matched by gPM+ clients’ gender. | A local Study Safety Committee that comprised 3 Jordanian health professionals was formed to monitor any adverse events that occurred during the trial. | 74.9% retention at 12 months. Although clients in gPM+ had greater reductions in depression at 3 months, at 12 months there were no significant differences between gPM+ and ECAU on depression and anxiety and other secondary outcomes except positive parenting. |
| de Graaff, Anne M., et al (2020); Netherlands | Adult Syrian refugees with elevated psychological distress resident in Rotterdam in Netherlands  60 (30 in treatment arm); 60% women; 38.1±12.2 | A single-blind pilot RCT using mixed-methods | Elevated psychological distress  (K10 >15); and reduced psychosocial functioning (>16 on WHODAS) | acute medical conditions, imminent suicide risk, expressed acute needs or protection risks, indications of severe mental disorders or cognitive impairment | Symptoms of depression and anxiety at 3-month follow-up. | Psychosocial functioning; symptoms of PTSD, and self-identified problems | Care As Usual (CAU): other mental health services available to Syrian refugees in the Netherlands. | Individual-based PM+ delivered face-to-face by 8 lay helpers. Clients received 5 sessions of PM+ & CAU in 5 consecutive weeks. Each session took 90 minutes | Helpers were  Peers (Syrian refugees) with at least high school  education and (professional) background in education, social  work or related field and a Certificate of Conduct. Trained for 8 days. | Helpers received weekly face-to-face group supervision by PM+ trainers/ supervisors. | 86.7% retention at 3 months. Significant differences in favour of PM+/CAU group were found for primary outcome and psychosocial functioning, PTSD symptoms, self-identified problems. |
| de Graaff, Anne M., et al (2023); Netherlands | Adult Syrian refugees with elevated psychological distress and reduced psychosocial functioning  206 (103 in treatment arm); 38% women; mean age 37 years | A single-blind RCT | Elevated psychological distress  (K10 >15); and reduced psychosocial functioning (>16 on WHODAS) | acute medical conditions, imminent suicide risk, expressed acute needs/protection risks, indications of severe mental disorders or cognitive impairment and ongoing treatment in specialized mental healthcare. | Symptoms of depression and anxiety at 3-month follow-up. | Psychosocial functioning; symptoms of PTSD, and self-identified problems | Care As Usual (CAU): other mental health services available to Syrian refugees in the Netherlands. | Individual PM+ (with option of in person or video call) delivered by lay helpers. Clients received 5 sessions of PM+ & CAU in 5 consecutive weeks. Each session took 90 minutes | Peer helpers (high school education, in social work or teaching) trained for 8 days followed by a practice case. | Helpers received weekly group supervision from mental health professionals. To evaluate treatment fidelity, helpers completed a checklist  addressing requisite PM+ components for each session. | At 3-month follow-up, PM+/CAU had greater reductions on psychological distress relative to CAU. PM+/CAU also showed greater reductions on depression, anxiety, PTSD symptoms, and self-identified problems but not on impairment. |
| **Author, year & country** | **Population; sample size; % female; age ±SD** | **Study design/**  **methods** | **Inclusion criteria** | **Exclusion criteria** | **Primary outcomes & follow-up time** | **Secondary outcomes** | **Control type** | **Intervention description** | **Intervention training & delivery** | **Supervision & quality control** | **Findings** |
| Rahman, Atif, et al (2016); Pakistan | Adult primary care attendees with high levels of both psychological distress and functional impairment  346 (172 in treatment arm); 78.9% women; 33.0±11.8 | A single-blind individual RCT | A score of ≥3 or on a screening questionnaire for CMDs (GHQ) and ≥17 for functional impairments (WHODAS 2.0) | Imminent risk of suicide, severe mental disorder, or severe cognitive impairment. | anxiety and depression symptoms (HADS), independently measured at 3 months follow up | posttraumatic stress symptoms, functional impairment, personally identified problems, and symptoms of depressive disorder (PHQ9) | Enhanced Care as Usual: clients and accompanying relatives seen at least once by primary providers. Clients given an option of repeated consultation. | Individual PM+ delivered face to face by 9 lay health workers. Clients received 5 weekly sessions of PM+. Each session took 90 minutes | Lay helpers (12-16 years of education, and no prior training in MH) were trained for 8 days by local MH specialists (who had been trained by a master trainer for 6 days) followed by 3 practice cases | Helpers supervised in 2 groups on a weekly basis (2 hours) by local supervisors (who were themselves supervised monthly by a master trainer via skype – 1 to 2 hours). | At 3-month follow-up, PM+ group had greater reductions on anxiety and depression (HAD) posttraumatic stress symptoms, functional impairment, personally identified problems and symptoms of depressive disorder (PHQ9). |
| Khan, M. N., et al (2019); Pakistan | Women aged ≥18 years, referred for screening based on the judgment of their health workers that they were psychologically distressed  119 (54 in treatment arm); 100% women; ≥18 years | cluster randomized controlled feasibility trial | A score of >2 or on a screening questionnaire for CMDs (GHQ) and >16 for functional impairments (WHODAS 2.0) | Imminent suicide risk, severe cognitive impairment, or mental disorder. | Symptoms of anxiety and depression (HADS) at 7 week follow up | Symptoms of depression (PHQ9), PTSD symptoms, general psychological profile, levels of functioning, generalized psychological distress. | Enhanced Usual Care (EUC): HW received training in primary care referral pathways for treatment of CMDs. | Group PM+ delivered face-to-face by female lay helpers. Participants received 5 weekly sessions of gPM+. Each session took 120 minutes | 3 Lay helpers (16 years of education & no prior training in MH) and 3 lay supervisors trained for 6 days followed by 4 weeks of practice cases. Each gPM+ had 6 clients. | Weekly group supervision via skype (2-3 hours). | gPM+ found acceptable and feasible delivered by lay-helpers. Outcome evaluation found significant improvements in depression, anxiety, general psychological profile, and functioning. PTSD and depressive disorder symptoms showed a trend in favor of intervention. |
| Hamdani, Syed Usman, et al (2020); Pakistan | Primary care attendees with high levels of psychological distress  346 (172 in treatment arm); 78.9% women; 33.0±11.8 | RCT | A score of >2 or on a screening questionnaire for CMDs (GHQ) and >16 for functional impairments (WHODAS 2.0) | Imminent risk of suicide, severe mental disorder, or severe cognitive impairment. | Cost-effectiveness of the PM+ intervention compared with  EUC for CMDs at 3 month follow up | Symptoms of depression (PHQ9), PTSD symptoms, general psychological profile, levels of functioning, generalized psychological distress. | Enhanced Care as Usual: clients and accompanying relatives seen at least once by primary providers. Clients given an option of repeated consultation. | Individual PM+ delivered face to face by 9 lay health workers. Clients received 5 weekly sessions of PM+. Each session took 90 minutes | Lay helpers (12-16 years of education, and no prior training in MH) were trained for 8 days by local MH specialists (who had been trained by a master trainer for 6 days) followed by 3 practice cases | Helpers supervised in 2 groups on a weekly basis (2 hours) by local supervisors (who were themselves supervised monthly by a master trainer via skype – 1 to 2 hours). | Cost of delivering PM+ per participant was US$163.14 (international trainer and supervisor), and US$35 (local trainer). PM+ was more effective but also more costly than EUC in reducing symptoms of anxiety, depression and improving functioning in adults. |
| **Author, year & country** | **Population; sample size; % female; age ±SD** | **Study design/**  **methods** | **Inclusion criteria** | **Exclusion criteria** | **Primary outcomes & follow-up time** | **Secondary outcomes** | **Control type** | **Intervention description** | **Intervention training & delivery** | **Supervision & quality control** | **Findings** |
| Hamdani, Syed Usman, et al (2021); Pakistan | Adult outpatient department attendees, referred for psychological support by psychiatrists  192 (96 in treatment arm); 68% women; 34.5±10.5 | two arm, single-blind individual RCT | Adults (18-60 years), a score of >2 or on a screening questionnaire for CMDs (GHQ) and >16 for functional impairments (WHODAS 2.0) | imminent risk of suicide, severe mental disorder, or severe cognitive impairment | symptoms of anxiety and depression (HADS) and functional impairment (WHODAS) 20 weeks after baseline assessment | depressive symptoms (phq9), PTSD symptoms, personally identified problems, perceived social support | Treatment As Usual (TAU): Initial assessment by trainee psychiatrists and expert consultation from psychiatrists | Individual PM+ delivered face to face by masters degree psychologists. Clients received 5 weekly sessions of PM+/TAU. Each session took 90 min | PM+ providers received 8 days training from a master trainer | fortnightly supervision meetings with the master trainer. | Follow up rate of 67% at primary endpoint. PM+/TAU group had significantly greater reductions in symptoms of anxiety and depression and in functioning. |
| Sangraula, M., et al (2020); Nepal | Adults with high levels of psychological distress and functional impairment  121 (61 in treatment arm); 83% women; PM+=46.7  EUC=49.3 | two-arm cluster RCT | Adults ≥18 years, fluent in Nepali, a score of >2 or on a screening questionnaire for CMDs (GHQ) and >16 for functional impairments (WHODAS 2.0). | A score of 16 on AUDIT, suspected severe psychiatric disorders, and respondents not able to comprehend or answer the consent and/or demographic questions coherently. | depression symptoms assessed using the PHQ-9 administered at baseline and 8–8.5 weeks post-baseline | daily functioning; psychological distress; PTSD symptoms; personalized outcomes; suicidality, perceived social support | EUC: psycho-education, providing referral options to primary care services with workers trained in the mhGAP-IG | Group PM+ delivered face-to-face by lay helpers. Participants received 5 weekly sessions of gPM+. Each gPM+ had 6-8 people and helpers were gender matched with clients. Sessions took 2.5 to 3 hours. | Helpers (10 years of education, >25 years) were trained for 20  Days. Group ‘helpers’ received a basic 2-day training on assisting facilitators : logistics and childcare | Clinical supervisors attended at least 2 of the 5 sessions per PM+ group and used the fidelity checklist as a tool to rate the skills of the facilitators. | Average attendance of 4 out 5 PM+ sessions. PM+ found feasible and acceptable. Though study not powered to assess effectiveness, for all the 5 key outcome measures, the mean improvement was larger in the gPM+ group than EUC group. |
| Jordans, Mark JD, et al (2021); Nepal | Adults with high levels of psychological distress and functional impairment in a disaster-prone setting  611 (319 in treatment arm); 82% women; 18-91 years | cluster RCT | Adults ≥18 years, screening positive for psychological distress and functional impairment. | Presence of a severe mental disorder (e.g., psychosis), cognitive impairment, or harmful alcohol use. | psychological distress assessed with the General Health Questionnaire  at 3 month follow up | depression symptoms, posttraumatic stress disorder (PTSD) symptoms, “heart–mind” problems, social support, somatic symptoms, and functional impairment | EUC: family psycho-education meeting with a referral option to primary care providers trained in mental healthcare | Group PM+ delivered face-to-face by lay helpers. Participants received 5 weekly sessions of gPM+. Helpers were matched with the clients’ identified gender. Each session took 2.5 hours | Lay helpers (at least high school education, living in the same community, and no prior training in MH) were trained for 20 days followed by practice sessions. 1 PM+ group was formed per ward in community locations. | Face-to-face group supervision was provided weekly. The 2 supervisors were trained psychologists and experienced counselors. | Results show initially moderate treatment effects and smaller benefits at 3-month follow-up in reducing psychological distress. Greater reductions were also seen for depressive symptoms, heart mind problems (local idiom for distress). No differences were seen for other secondary outcomes. |
| **Author, year & country** | **Population; sample size; % female; age ±SD** | **Study design/**  **methods** | **Inclusion criteria** | **Exclusion criteria** | **Primary outcomes & follow-up time** | **Secondary outcomes** | **Control type** | **Intervention description** | **Intervention training & delivery** | **Supervision & quality control** | **Findings** |
| Dawson, Katie S., et al (2016); Kenya | women affected by urban adversity and gender-based violence  70 (35 in treatment arm); 100% women;  PM+=33.3  ETAU=37.6 | Feasibility RCT | Being female, over 18 years of age, and a score of three or above on the General Health Questionnaire and 17 WHODAS | Those at risk of ending their life or displayed severe mental disorder or severe cognitive impairment. | measure of general psychological distress - one to two weeks after the scheduled 5th session of intervention (or approximately 6 weeks later for the control group) | Functioning and disability (WHODAS); Gender-based violence; Stressful life events; Post-traumatic stress  symptoms | Enhanced Treatment as Usual (ETAU): receiving care from primary care clinicians at one of 3 local facilities from primary care nurses who had received 1 day training in MH | Individual PM+ delivered face to face by 23 community health workers (all women).  Clients received 5 weekly sessions of PM+. Each session took 90 min | Lay helpers (CHWs) were trained for 8 days by a master trainer and 3 Kenyan psychologists who would provide supervision to the CHWs. This was followed by 4 weeks of practice cases- (3) | CHWs were supervised on a weekly basis by one of the 3 local supervisors who were clinical psychologists. Local supervisors were supervised weekly to fortnightly (1-2 hours). | PM+ was found to be feasible and acceptable. The study was not powered to identify effects and accordingly did not identify effects on the primary outcome measure of general psychological distress |
| Bryant, Richard A., et al (2017); Kenya | women with a history of gender-based violence in urban in Nairobi Kenya  421 (209 in treatment arm); 100% women;  PM+=35.2  EUC=35.9 | single-blind, parallel RCT | A history of GBV, score of 3 or above on the GHQ-12, and a score of 17 or above on WHODAS | imminent plans of suicide,  psychotic disorders, severe cognitive impairment. | psychological distress(GHQ-12) assessed at 3 months after treatment. | impaired functioning, symptoms of posttraumatic stress, personally identified problems, stressful life events, and health service utilization. | EUC: provide by 6 community nurses at clinics in the area. Nurses were given 2-day non-specific training in counselling skills and PFA. | Individual PM+ delivered face to face by 23 community health workers (all women).  Clients received 5 weekly sessions of PM+. Each session took 90 min | CHWs were trained for 8 days (64 hours). Two local supervisors (psychologists) were also trained. CHWs also received 1 day training on PFA. | During the trial, CHWs received 2 hours of group weekly supervision (5 CHWs per group) from a local supervisor who received 1.5 hours of weekly training and mentoring in supervision. | PM+ group had significantly greater reductions on psychological distress. Similar patterns were seen for functioning, PTSD symptoms, and personally identified problems. |
| Van't Hof, Edith, et al (2018); Kenya | 27 key informants six women who completed PM+, six community health volunteers  who delivered the intervention, seven people with local decision-making power, and eight project staff involved in the PM+ trial. | Qualitative exploration | Participants, intervention delivery agents, investigators or stakeholders involved in a previous PM+ RCT in Kenya | NA | acceptability of PM+ and possible barriers and facilitators of implementing PM+ as perceived by different stakeholders involved in an RCT in Kenya | NA | NA | NA | NA | NA | Participants and CHVs reported the positive impact PM+ had made on their lives. Potential structural and psychological barriers to scale up were identified. The sustainability of CHVs as unsalaried, volunteer providers was mentioned by most interviewees as the main barrier to scaling up the intervention. |
| **Author, year & country** | **Population; sample size; % female; age ±SD** | **Study design/**  **methods** | **Inclusion criteria** | **Exclusion criteria** | **Primary outcomes & follow-up time** | **Secondary outcomes** | **Control type** | **Intervention description** | **Intervention training & delivery** | **Supervision & quality control** | **Findings** |
| Nyongesa, Moses Kachama, et al (2022); Kenya | Young people living with HIV with mild-to-moderate symptoms of CMDs  70 (35 in treatment arm); 65.7% women;  18 to 24 years | mixed-method formative research employing both qualitative and quantitative methods | 18–24 years.  living with HIV,  having mild or moderate symptoms of CMDs, consent for participation, and access to a mobile phone | Risk of suicide, severe mental, neurological or substance use disorders | Depressive and anxiety symptoms (PHQ9 and GAD7) – within 2 weeks after the last session | Quality of life (FAHI) and perceived social support (Social provisions scale). | Waitlist who received EUC: referral to the HIV clinics with participants consent but with no follow up. A 5–10-minute weekly telephone call to check their wellbeing. Waitlist participants who completed follow up received adapted PM+ | Individual PM+ delivered over the phone by 4 trained lay helpers. Intervention arm received 10 weekly sessions of PM+ (45 min each) | 4 Lay helpers (at least high school education but no prior training in MH) received 3 weeks of training (including 10 days of infield practice) by a clinical psychologist. | Helpers received weekly supervision. Supervisor observed at least 3 randomly selected sessions from each helper. An additional meeting (1.5 to 2 hours) held with helpers & trainer every fortnight for additional support | PM+ found contextually appropriate, acceptable, and feasible for mobile phone delivery. 69% retention. Preliminary feasibility data indicated that the adapted PM+ has the potential of reducing CMDs among YLWH. |
| Dowrick, Christopher, et al (2022); UK | distressed and functionally impaired asylum seekers and refugees | three-arm Feasibility  RCT: Control, Individual PM+ and Group PM+ | AS&Rs being ≥ 18 years of age, experiencing emotional and practical difficulties, being registered with a GP in Liverpool City Region, and having the ability to converse in English. | AS&Rs new to initial accommodation, or currently receiving psychological therapy, or experiencing severe mental disorder(s) or cognitive impairment | anxiety and depressive symptoms at 3 months, measured using the HADS at 3 and 6 months | subjective well-being, functional status, progress on identified problems, post-traumatic stress disorder, depressive disorder and service usage. | Usual care and support offered by participating NGOs. | Individual PM+ and Group PM+ delivered by 12 trained lay therapists with lived experience of the asylum process. Intervention arm received 5 weekly sessions of about 90 min each of PM+ | 2 wellbeing mentors and supervisors received 5 days of training from PM+ master trainers. Lay therapists received 8 days of training followed by practice cases and competency assessment. | mentors received monthly supervision (1.5 to 2 hours) and complemented with email and telephone discussions as necessary. Lay therapists monitoring continued throughout the trial | Trial was open for recruitment for 3.5 months but was interrupted by COVID-19. 11 people were randomised. 8 (73%) were successfully followed for 3 months and 7 (64%) for 6 months. |
| Knefel, Matthias, et al (2022); Austria: | Dari-speaking adult Afghan asylum seekers or refugees which were seeking help for mental health problems  51 (26 in treatment arm); 49% women;  34.3±13.6 | prospective, single-center, assessor  masked, randomized, two-group superiority trial | being on a wait list for mental health treatment and elevated psychological distress based on a screening questionnaire for CMDs | acute suicidality, severe mental disorder or severe cognitive impairment and current trauma-focused treatment. | general health (GHQ-28) 1 week post intervention | distress by PMLD, Complex PTSD symptoms, quality of life, self-identified problems, and integration | TAU: All other health services available to Afghan refugees in Austria. | Individual face-to-face PM+ delivered by clinical psychologists. Clients got PM+/TAU – 6 weekly 90 min sessions. | The psychologists were trained by two WHO PM+ master trainers | Regular supervision was provided to ensure psychologists’ adherence to the protocol. | 42% attrition. PM+ was effective in reducing general health problems in Afghan refugees and  might be considered as a first-line intervention. |
| **Author, year & country** | **Population; sample size; % female; age ±SD** | **Study design/**  **methods** | **Inclusion criteria** | **Exclusion criteria** | **Primary outcomes & follow-up time** | **Secondary outcomes** | **Control type** | **Intervention description** | **Intervention training & delivery** | **Supervision & quality control** | **Findings** |
| Knefel, Matthias, et al (2022); Austria: | Mental Health Professionals: psychologists, psychotherapists, and psychiatrists  59; 88.8% women;  35.9±14.4 | Online exploration | judgment sampling approach | NA | perception of the usability of adapted Problem Management Plus | NA | NA | NA | NA | NA | MHPs had a positive view on the intervention but emphasized the importance  of situation-specific adaptations to the structure of the manual. The most favoured specific  strategies were ‘managing stress/slow-breathing’, the ‘tree of capabilities’, and the ‘riding the anger’ exercise |
| Zhang, Hong, et al (2020); China | Cases of multiple myeloma who presented depressive symptoms  80 (40 in treatment arm); 28 women;  Mean age 63.2 | RCT | confirmation of multiple myeloma; clients who were not treated with induction therapy | coexisting serious diseases such as cardiac, respiratory, liver, and renal dysfunctions or severe malnutrition | anxiety and depression symptoms, independently measured at 3 months with the Hospital Anxiety and Depression Scale | psychological outcome profiles (PSYCHLOPS), measure of functioning (WHODAS 2) | Controls underwent investigation without management | Individual face-to-face PM+ delivered by 8 nurses. Clients received five 90-minutes weekly sessions. | Prior to initiation of the intervention, they were trained as assistants and underwent supervision | Not mentioned | PM+ group showed greater reductions in psychological profile scores, anxiety and depression. |
| Spaaij, Julia, et al (2022); Switzerland | Syrian refugees and asylum seekers experiencing elevated levels of psychological distress  59 (31 in treatment arm); 50.8% women;  PM+ = 39.55  ETAU = 40.27 | single-blind pilot RCT | Syrian refugees who arrived in Switzerland after the outbreak of the Syrian civil war in 2011; 18 years or older; Arabic-speaking; elevated psychological distress and impaired psychosocial functioning | severe cognitive impairment; severe mental disorders; acute risk of suicide; being under guardianship; and inability to follow the study procedures. | Psychological distress; health and disability across six dimensions Qualitative evaluation of feasibility and acceptability was explored from several key informant groups | Symptoms of depression and anxiety; Previous exposure to potentially traumatic events; post-migration stressors symptoms of PTSD, information on health and other service use. | ETAU: clients given a booklet explaining the Swiss healthcare system in Arabic and told to contact their GP in they needed MH assistance and could contact research team anytime. | Individual face-to-face PM+ delivered by 13 lay helpers. Clients received five 90-minutes weekly sessions. | Helpers received 8-day training by a Master trainer and a PM+ trainer. | Helpers received continuous supervision – weekly initially and later monthly | 67.8% retention and mean intervention attendance of 3.94 sessions. Findings indicate that trial procedures and PM+ was feasible, acceptable and safe. |
| **Author, year & country** | **Population; sample size; % female; age ±SD** | **Study design/**  **methods** | **Inclusion criteria** | **Exclusion criteria** | **Primary outcomes & follow-up time** | **Secondary outcomes** | **Control type** | **Intervention description** | **Intervention training & delivery** | **Supervision & quality control** | **Findings** |
| Qi, Aili, Fatao Wang, and Tiwang Cao (2023); China | parents of children with ASD  73 (36 in treatment arm); 54.8% women; | Not mentioned | parents of children with ASD diagnosed by the hospital; those without serious respiratory diseases, cardiovascular & cerebrovascular diseases; those without the habit of physical exercise | those who did not practice Tai Chi according to the teaching requirements; those who participated in less than 90% of the training course; and those who quitted midway. | Social anxiety (Social Anxiety Scale for Children) | Parenting Stress Index-Short Form and Social Support Rate Scale | The control group did not receive any psychological intervention. | Intervention arm received individual PM+ & Tai Chi training in addition to routine health education. Intervention was online (live streaming) regularly 14 times in 7 weeks, 2 times each week, 40 minutes each time. | Not reported | Not reported | Treatment arm had significantly higher decreases in social anxiety, parenting stress, distress and dysfunctional parent-child interaction. They also had higher improvement in social support. |
| Nemiro, A., Van’t Hof, E., & Constant, S. (2021); Ethiopia, Syria, and Honduras | NA | Case studies field report | NA | NA | NA | NA | NA | PM+ | NA | NA | Case studies show PM+ is relevant and appropriate for use in various humanitarian settings. Having a robust supervision system is essential along with practicing the intervention before delivery, dedicated staff, adequate time for training. |
| Akhtar, Aemal, et al (2021); Jordan and Turkey | Adult Syrian refugees (≥18 years old) living in Jordan in camp or community locations.  103 participants in the RQA. | Qualitative Interviews following the DIME model | Purposeful sampling | NA | adapt a scalable psychological intervention for Syrian refugees in urban and camp settings. | NA | NA | NA | NA | NA | Based on the results a total of 82 changes were proposed across the intervention manual, training, supervision, and implementation protocols: ranging from minor amendments to terminology to broader changes to how metaphors, stories are presented during intervention. |
| **Author, year & country** | **Population; sample size; % female; age ±SD** | **Study design/**  **methods** | **Inclusion criteria** | **Exclusion criteria** | **Primary outcomes & follow-up time** | **Secondary outcomes** | **Control type** | **Intervention description** | **Intervention training & delivery** | **Supervision & quality control** | **Findings** |
| Perera, Camila, et al (2020); Colombia. | displaced Venezuelans and Colombians | a process for culturally  adapting low-intensity interventions | NA | NA | NA | NA | NA | NA | NA | NA | The proposed four-step process offers a useful guide for how to adapt low-intensity psychological  intervention within humanitarian settings. |
| Coleman, Sarah F., et al (2021); Rwanda, Peru, Mexico and Malawi | community and primary care settings in Rwanda, Peru, Mexico and Malawi | Case studies | NA | NA | To describe the cross-site process of adapting PM+ for implementation | NA | NA | NA | NA | NA | Although each site is unique, sites employed similar adaptation methods that have applicability across other settings |
| Bryant Richard A et al (2022); Australia | Adults in Australia distressed by COVID-19;  n=240, 120 intervention arm  83.8% female  age intervention - 37.3±12.8, EUC -36.6±12.9 | proof-of-concept trial  (single-blind, parallel, RCT) | (a) adult (18 years or older), (b) score ≥3 on the GHQ-12, and (c) adequate English language comprehension. | Self-report on current psychosis, imminent suicidal risk, current substance dependence, current psychotherapy,  or no internet-based access for videoconferencing | Severity of anxiety and depressive  symptoms at 1 week post-treatment, 2 months (primary  follow-up time point) and 6 months | Worry,  Sleep difficulties, Anhedonia,  Pandemic-related worries | EUC comprised emailing participants a resource  package comprising handouts detailing the strategies taught in the  intervention. | adapted Group PM+ delivered to groups of four participants on a videoconferencing platform. It consisted of 6 × 60-min sessions conducted over a period of 8 weeks | The facilitators were trained by  Master trainer over 8 days, and each  led a full practice group program  under supervision by master trainer | Protocol adherence - sessions were recorded, and two  independent psychologists rated 20% of sessions using a checklist | Relative to EUC, at 2 months’ PM+ group showed greater reduction  on anxiety and depression.  The effects were maintained at 6  months. There were also greater reductions of worry, anhedonia, COVID-19-related fears, and contamination fears. |
| Galea, Jerome T et al (2023) USA | 5 First year Master of Social Work students volunteering at the University of South Florida 'Bridge' free Clinic | Piloted experiential learning report | N/A | N/A | PM+ training allowed student practitioners to acquaint themselves with therapeutic skills (e.g. breathing exercises, identifying social  supports) | Improving the mental health of bridge patients receiving PM+ | N/A | Person instruction (i.e., breakdown of skills, roleplays, and live feedback) self-study utilizing the PM+ training manual,  screening patients to receive PM+, and implementing the  intervention. | 5 in person training days. After 3 days of in-person training, students were able to deliver PM+ competently. | Regular supervision during implementation without affecting the program’s integrity and delivery. | Students agreed that the training provided a new clinical skill and appreciated that they did not need a clinical background to learn PM+ intervention. |
| **Author, year & country** | **Population; sample size; % female; age ±SD** | **Study design/**  **methods** | **Inclusion criteria** | **Exclusion criteria** | **Primary outcomes & follow-up time** | **Secondary outcomes** | **Control type** | **Intervention description** | **Intervention training & delivery** | **Supervision & quality control** | **Findings** |
| Dozio E., Dill Ann S. & Bizouerne C. (2023); Central African Republic | Internally displaced persons (IDPs) in Bangui n=946, no control arm  84% female mean age, SD : 35.7 ± 9.31 | Non controlled non randomized (pre-post) | Beneficiaries (adults) of Income Generating Activities (IGAs) programme in the population of PK5 (Point Kilometer 5) in the neighbourhoods of the 3rd arrondissement of Bangui and in the Boeing locality in the commune of Bimbo. | People with severe mental illness,  risk of suicide or cognitive impairments | General wellbeing, Self-reported wellbeing, Functioning; Posttraumatic stress disorder,  Baseline, endline (after 5 weeks), and post intervention (1 month after endline) | None | No Control arm | PM+ adapted for group use (max 10 people per group), 5 weekly sessions of approximately 2hours each | Each group facilitated by two psychosocial workers who received a 2-week initial training (including group facilitation techniques) | continuous supervision of the trained psychosocial  workers by an expert psychologist. | Results showed a statistically significant reduction of the PTSD symptoms, functional impairment, personally identified problems and an improvement of general wellbeing between baseline and endline scores. |
| Mediavilla R et al. (2023); Spain | HealthCare Workers employed by the Department of Health with psychological distress; n=232 (115 in treatment arm); 86% women; 37.5±10.3 ( Age for control - 37.1±10.4, Age for intervention - 37.9+10.1) | A multicentre, parallel-group, analyst-blinded RCT | a) Employed by either the Madrilenian or the Catalan Department of Health (doctors, psychologists, nurses, nursing technicians, orderlies, and administrative staff) b) Being psychologically distressed (K10 ≥16) c) Able to read Spanish or Catalan | a) Having an acute medical condition that required immediate hospitalization b) Imminent risk of suicide or self-harm or risk of harming others c) Severe mental disorder; severe cognitive impairment d) Having initiated, stopped or significantly modified pharmacotherapy or standardized psychological treatment in the previous 8 weeks | Self-reported anxiety and depression symptoms 2 months after the full stepped-care programme (comprised two scalable psychological interventions: a guided stress management course based on the SH+ booklet called Doing What Matters in  Times of Stress and PM+) | Anxiety, depression, and PTSD symptoms at weeks 7, 13, and 21 | Received care as usual enhanced with Psychological First Aid (eCAU) | Participants received eCAU; then offered the stepped-care programme (comprised two scalable psychological interventions: a guided stress management course based on the SH+ booklet called Doing What Matters in Times of Stress (DWM) and PM+); Both interventions had an online format, each took 5–6 weeks to be delivered  Adapted PM+ shortened from 90 to 60 mins to fit better within a work setting  and tailored case examples to HCW | The intervention providers were mental health providers who received specific preparation (~50 hours); DWM was provided as guided self-help and included weekly phone-based or message-based contacts lasting 15 min; PM+ sessions were shortened from 90 to 60 min to fit better within a work setting; The criterion for stepping up to PM+ was reporting significant levels of psychological distress (K10≥16) 5–7 days after the DWM (week 7). | Attendance of weekly supervision sessions by intervention providers while the trial was ongoing (~30 hours); The trainers/supervisors were psychiatrists and clinical psychologists instructed by the intervention developers. | 79.3% retention. Three in four participants stepped up to PM+ (n=86, 75%); Overall decrease in anxiety and depression symptoms at the primary endpoint (week 21) was larger in the intervention arm compared with  the eCAU. The decrease was also larger in the intervention arm relative to eCAU at all remaining time points, that is, after DWM or week 7  and after PM+ or week 13 and across all secondary outcomes, except for post-traumatic stress symptoms after the DWM |
| **Author, year & country** | **Population; sample size; % female; age ±SD** | **Study design/**  **methods** | **Inclusion criteria** | **Exclusion criteria** | **Primary outcomes & follow-up time** | **Secondary outcomes** | **Control type** | **Intervention description** | **Intervention training & delivery** | **Supervision & quality control** | **Findings** |
| Gebrekristos, F., Eloul, L., & Golden S. (2021); Ethiopia | Eritrean refugees primarily survivors of torture or severe trauma experiences | Field report on PM+ implementation | Meeting the correct level of distress into the intervention for PM+ intervention | Severe mental disorder | NA | NA | NA | NA | The trainers first provided PM+ training to 27 Center for Victims of Torture counseling staff. The second stage was to train two cohorts of partner organisation staff. Training days were 7 hours (cohort 1), and helpers did not have additional work duties during the two-week training and 5hours (cohort 2) and helpers had additional duties during the two-week training period. | Intensive supervision was required for the first course of clients which required significant time investment, with 90–120 minutes per session and 20–30 minutes for preparation and debriefing. Live supervision was provided for all five sessions with the  helpers’ first clients. | Helpers demonstrated notable improvement between the pre- and post-training role-play assessments, particularly in their capacity to maintain a therapeutic presence. Most helpers were able to understand and adopt nonverbal skills such as facial expression, posture and vocal tone. However, verbal communication competency varied, and required more coaching to implement normalization |
| Musotsi P et al., 2022; Iraq | IDPs, returnees and host community adults with emotional distress and/or functional impairment; n=290 | non controlled non randomized (pre-post) | 18 years and above of age. | a) > 18 years  b) scored <17 on WHODAS  c) scored <3 on PHQ-9  d) had plans to end their lives within two weeks of the pre-assessment  e) had a severe mental health disorder  f) those judged to have a neurological or severe mental disorder, substance use disorder | Provide mental health and psychosocial support (MHPSS) to address the mental health needs of adults; WHODAS, PSYCHLOPS, and PHQ-9 used to assess for mental health distress. | NA | NA | Individual PM+ was delivered over five weeks, with each  weekly session lasting 90 minutes.  Delivered at the location of the participant’s choice and by a lay counsellor of the same gender as the participants for cultural appropriateness. | Lay counsellors received 3 weeks of training. Four days were used for training on psychological first aid (PFA), enhanced with basic psychosocial support skills and 8 days for PM+ training in the classroom and the field. Also received positive parenting training for 2 days. | Lay counsellors underwent supervised in-field practice, offering 5 sessions of PM+ to three clients with less severe presentations.  Lay counsellors received weekly clinical supervision from trained professional psychologists. | Significant reduction in the mean PSYCHLOPS score, functionality, between pre-assessment and post-assessment |
| **Author, year & country** | **Population; sample size; % female; age ±SD** | **Study design/**  **methods** | **Inclusion criteria** | **Exclusion criteria** | **Primary outcomes & follow-up time** | **Secondary outcomes** | **Control type** | **Intervention description** | **Intervention training & delivery** | **Supervision & quality control** | **Findings** |
| Tay, Alvin Kuowei, et al., 2020;  Malaysia | Rohingya, Chin, and Kachin refugees living in Malaysia;  N=331 (114 PM+ clients);  72% men; mean age 30.8 years | single-blind RCT | (a) presence of at least one of the designated CMDs, (b) witnessed or experienced at least one traumatic event related to mass conflict; and (c) endorsed at least one ADAPT-related stressor on each scale of the relevant measure. | age lower than 18 years, overt evidence of intellectual disability or cognitive impairment, or manifestations of psychosis | Post Traumatic Stress Disorder, Complex PTSD, Major Depressive Disorder, the 5 scales of the Adaptive Stress Index , and a measure of resilience  6 weeks post treatment | Anxiety symptoms, and Persistent Complex Bereavement Disorder | PM+ (here referred to as Cognitive Behavioural Therapy - CBT) was evaluated as a comparator intervention to a primary intervention called Integrative Adapt Therapy - IAT). | CBT included 6 strategies – drawn from WHO PM+ with an additional component of cognitive reappraisal.  The strategies were delivered sequentially over 6-weekly sessions, each session 45 minutes. | CBT was delivered by trained lay counsellors. Eight days were devoted to training in IAT and the same period for CBT. Trainees then progressed to 8 weeks of field practice. Each counsellor was assessed for their competency | Treatment fidelity for all counsellors in both modalities of therapy was assessed by 2 independent assessors, each trained in either IAT or CBT. | Compared to CBT, IAT participants reported greater reductions in mental health symptoms, in all 5 adaptive stress areas (except the injustice domain), and a greater increase in resilience |
| Fuhr, D.C et al (2020); Turkey | Twenty-four stakeholders (including governmental officials, mental health providers, officials from  international/  national non-governmental organisations, conflict and health researchers) | Theory of change (TOC) workshop | External stakeholders who were not involved in developing and adapting PM+  in Turkey | NA | (a) investigate the use of ToC methodology in planning the scale up of PM+ for Syrian refugees in Turkey; (b) to explore  context-specific pathways of scaling up PM+ for Syrian refugees in Turkey; and (c) to identify barriers and facilitators to scale up. | NA | NA | NA | NA | NA | A ToC map was produced identifying three key elements of scaling up (the resource team; the innovation and the health system; and the user organisation) which are represented in three distinct causal pathways. Context-specific barriers related to the health system and the political environment were identified, and possible strategies for overcoming these challenges were suggested. |
| **Author, year & country** | **Population; sample size; % female; age ±SD** | **Study design/**  **methods** | **Inclusion criteria** | **Exclusion criteria** | **Primary outcomes & follow-up time** | **Secondary outcomes** | **Control type** | **Intervention description** | **Intervention training & delivery** | **Supervision & quality control** | **Findings** |
| Fuhr, D.C et al (2020); Multicountry (Turkey, the Netherlands, and Lebanon) | 15–24 stakeholders were invited per country, based on their expertise on the mental health system and policy in the country, knowledge on the provision of services including PM+ and EASE, and refugee mental health needs | ToC workshop in the 3 countries where PM+ and EASE are implemented for Syrian refugees | NA | NA | (a) to report findings of a cross-country  ToC map for scaling up PM+ and EASE for Syrian refugees; (b) to highlight cross-country barriers and facilitators to scale up; and (c) to suggest political and health  system changes to make scale up a reality | NA | NA | NA | NA | NA | Two distinct causal pathways for scale up were identified (a policy and financing pathway, and a health services pathway) which are interdependent on each other. A list of key assumptions and interventions which may hamper or facilitate the scaling up process were established. |
| McBride, K.A., et al (2023);  Multicounty  (USA, Greece, Sweden, Italy, Nigeria, Belgium, Ethiopia, Uganda, Rwanda, South  Africa, Burundi, Somalia and Somaliland) | Cohort 1: students beginning their first or second year of the clinical psychology doctoral programme (N=8) at the NSSR and already supervised by licensed professional within the state and eight trainees preidentified to become trainers of Cohort 1. Cohort 2: 16 trainees from SOS Children’s Villages International | Adaptation of PM+ for remote training and implementation | Appropriate candidates in Cohort 2 were selected based on a series of considerations, including: (a) potential need in the context, (b) pathways for referrals to specialized care, (c) some degree of previous experience and proven skills in the helping relationship and/or MHPSS interventions, (d) English language proficiency, (e) a stable internet connection and (f) availability to participate | NA | NA | NA | NA | NA | Pre-survey and assessment of trainees with a remote version of Enhancing Assessment of Common Therapeutic Factors (ENACT). The actor explains role plays which are followed by mock session interviews. Role-plays are prerecorded and then observed by trained raters who complete the ENACT scoring. | There was supervision of practice cases by the trainers. Supervision takes place in group format on Zoom and individual sessions have been held when necessary. Self-care check-ins and in-depth discussions about caring for yourself as a helper have been integrated into supervision. | Remote PM+ Training may be Feasible. Overall, the trainers were successful in delivering the training to the trainees. The majority of participants completed the training and were competently prepared to deliver the PM+ intervention. PM+ may be adapted for remote (online) training and, if supported with additional studies, could build workforce capacity in contexts in which there is limited in-person access. |
| **Author, year & country** | **Population; sample size; % female; age ±SD** | **Study design/**  **methods** | **Inclusion criteria** | **Exclusion criteria** | **Primary outcomes & follow-up time** | **Secondary outcomes** | **Control type** | **Intervention description** | **Intervention training & delivery** | **Supervision & quality control** | **Findings** |
| Perera et al., 2022; Venezuela | Venezuelan refugees and migrants and Colombian returnees; n=72 (39 in treatment arm); 36.1% women; 34.4±9.6 ( Age for control - 39.3±10.3, Age for intervention - 30.2±6.7) | Randomised wait-list controlled trial | a) >18 years  b) Venezuelan migrants, refugees or Colombian  returnees  c) Planned to stay in Saravena for at least six months  d) Scored ≤74 and >28 on the WHO-5 | a) Scored ≤ 28 in the WHO-5  b) Reported thoughts of suicide | Mental health and well-being measured by WHO-5, PSYCHLOPS, and WHOQOL-BREF | Context-specific factors influencing the implementation of PM+ and how they can be addressed, as perceived by those delivering and receiving PM+ through qualitative interviews | Wait-list controlled type - Received the intervention once a lay provider was available and received security clearance, after seven months of the first assessment | Individual PM+  5 weekly sessions each 90 mins | a) 4-day training of PM+ to Colombian Red Cross volunteers and Supervisors  b) All intervention providers had previous training in PFA | Hiring of 2 supervisors | Significant effect for the PM+ group across all outcome measures: WHO-5; WHOQOL-BREF Physical; WHOQOL-BREF Psychological; WHOQOL-BREF Social Relationships;  WHOQOL-BREF Environment |
| Rahman A. et al (2019) | Adult women aged 18-60 years in a conflict area.  612 (306 in PM+ arm); 100% women; 18-60 years. | single-blind, cluster, randomised, controlled trial | women scoring at least 3 on the General Health Questionnaire-12, and at least 17 on the WHO Disability Assessment Schedule. | Women with severe mental health disorders (e.g., psychotic disorders or substance dependence), severe cognitive impairment (e.g., severe intellectual disability), or at risk of imminent suicide were excluded. | combined symptom score of anxiety and depression, measured with the Hospital Anxiety and Depression Scale 3 months after intervention | PTSD symptoms, functional impairment, problems for which the person sought help, perceived social support, and prevalence of depressive disorders, all measured at the individual level. | EUC: feedback about the assessment results, the offer of psychoeducation- for themselves and accompanying family members and the opportunity to talk about their health with their LHW, and information about the options for seeking care for distress. | The group PM+  intervention consisted of five group sessions per week,  with approximately six to eight participants per group,  each session lasting for approximately 2 h (excluding  breaks) | Therapists, called facilitators, were local graduates with bachelor’s degrees without mental health-care experience. The facilitators received 7 days of intervention training by a master trainer. | Supervision of the facilitators was done through 2h of weekly group session via skype. In turn, the supervisors received 1·5 h of fortnightly supervision via Skype by the master trainer. | At 3 months, women in the intervention group had significantly lower mean total scores on the HADS than women in the control group. Individual HADS anxiety scores were also significantly lower in the intervention group than in the control group as were depression scores. No adverse events were reported in either group. |
