# Supplementary material for: A scoping review of the literature on the application and usefulness of the Problem Management Plus (PM+) intervention around the world
Source: BJPsych Open. 2024 Apr 23;10(3):e91. doi: 10.1192/bjo.2024.55 (PMC11060090; doi:10.1192/bjo.2024.55)
Supplement: Mwangala et al. supplementary material 3 — Mwangala et al. supplementary material [file S2056472424000553sup003.docx]

**Supplementary file 3| Mental health outcomes targeted by experimental studies**

|  | **Study detail** | **Primary outcome measures** | **Secondary outcome measures** |
| --- | --- | --- | --- |
|  | Acarturk, C., et al (2022); Turkey | Psychological distress measured by the Hopkins Symptoms Checklist (HSCL-25) at 3-month follow up. | PTSD Checklist for DSM-5 (PCL-5), the Psychological Outcomes Profiles Scale (PSYCHLOPS), and the Client Service Receipt Inventory (CSRI). |
|  | Akhtar, Aemal, et al (2021); Jordan | Psychological distress measured by the Hopkins Symptoms Checklist (HSCL-25) at 1-week post-assessment. | PTSD symptoms (PCL-5), self-identified problems (PSYCHLOPS), prolonged grief (PG-13), prodromal psychotic symptoms (Brief Prodromal Questionnaire-16) and Psychological distress in the children of participants (Paediatric Symptoms Checklist) |
|  | Bryant, Richard A., et al (2022); Jordan | Symptoms of depression & anxiety assessed at baseline, 6 weeks, and 3 months measured by the Hopkins Symptom Checklist-25 (HSCL-25) | functional impairment (WHODAS-12), PTSD symptoms (PCL-5), personally identified problems (PSYCHLOPS), prolonged grief symptoms (PG-13), prodromal psychotic symptoms (PQ-B), parenting behavior (APQ), and children’s self-reported mental health (Paediatric symptoms checklist. |
|  | Bryant, Richard A., et al (2022); Jordan | Symptoms of depression & anxiety assessed at 12 months measured by the Hopkins Symptom Checklist-25 (HSCL-25) | functional impairment (WHODAS-12), PTSD symptoms (PCL-5), personally identified problems (PSYCHLOPS), prolonged grief symptoms (PG-13), prodromal psychotic symptoms (PQ-B), parenting behavior (APQ), and children’s self-reported mental health using Paediatric symptoms checklist (PSC), and Traumatic events checklist to measure exposure to traumatic events. |
|  | de Graaff, Anne M., et al (2020); Netherlands | Symptoms of depression and anxiety at 3-month follow-up (Hopkins Symptom Checklist; HSCL-25) | functional impairment (WHODAS-12); symptoms of PTSD (PCL-5), self-identified problems (PSYCHLOPS), and Client Service Receipt Inventory (CSRI). |
|  | de Graaff, Anne M., et al (2023); Netherlands | Depression/anxiety combined (Hopkins Symptom Checklist; HSCL-25) at 3-month follow-up. | functional impairment (WHODAS 2.0), PTSD symptoms (PCL-5), and self-identified problems (PSYCHLOPS) |
|  | Rahman, Atif, et al (2016); Pakistan | Anxiety and depression symptoms (HADS), independently measured at 3 months follow up | PTSD symptoms (PCL-5), functional impairment (WHODAS 2.0), progress on problems for which the person sought help (PSYCHLOPS), and symptoms of depressive disorder (PHQ-9), stressful life events (a modified version of the Life Events Checklist) |
|  | Khan, M. N., et al (2019); Pakistan | Individual psychological distress, measured by levels of anxiety and depression on the HADS at 7th week after baseline. | Symptoms of depression (PHQ-9), PTSD symptoms (PCL-5), general psychological profile (PSYCHLOPS), levels of functioning (WHODAS 2.0) and generalized psychological distress (GHQ) |
|  | Hamdani, Syed Usman, et al (2020); Pakistan | Effectiveness was measured using the Hospital Anxiety and Depression Scale (HADS) at 3 months post-intervention. Cost-effectiveness analysis was performed as incremental costs (measured in Pakistani rupees, PKR) per unit change in anxiety, depression, and functioning scores. |  |
|  | Hamdani, Syed Usman, et al (2021); Pakistan | Symptoms of anxiety and depression (HADS) and functional impairment (WHODAS 2.0) at 20 weeks after baseline. | Depressive symptoms (PHQ-9), PTSD symptoms (PCL-C), personally identified problems (PSYCHLOPS), perceived social support (Multidimensional Scale of Perceived Social Support) |
|  | Sangraula, M., et al (2020); Nepal | Depression symptoms (PHQ-9) administered at baseline and 8–8.5 weeks post-baseline | Daily functioning (WHODAS 2.0); psychological distress (heart and mind screener); PTSD symptoms (PCL-5); personalized outcomes; suicidality, perceived social support (Multidimensional Scale of Perceived Social Support); Client Services Receipt Inventory (CSRI) |
|  | Jordans, Mark JD, et al (2021); Nepal | Psychological distress assessed with the General Health Questionnaire at 3 month follow up | Depression symptoms (PHQ-9), PTSD symptoms (PCL), “heart–mind” problems, social support (Multidimensional Scale of Perceived Social Support), somatic symptoms, and functional impairment (WHODAS 2). |
|  | Dawson, Katie S., et al (2016); Kenya | Measure of general psychological distress - one to two weeks after the scheduled 5th session of intervention (or approximately 6 weeks later for the control group) – GHQ-12 | Functioning and disability (WHODAS); Gender-based violence (WHO-VAW); Stressful life events; Post-traumatic stress symptoms (PCL-5). |
|  | Bryant, Richard A., et al (2017); Kenya | Psychological distress(GHQ-12) assessed at 3 months after treatment. | Impaired functioning (WHODAS), symptoms of posttraumatic stress (PCL), personally identified problems (PSYCHLOPS), stressful life events (LEC), and health service utilization (service receipt inventory [SRI]), gender-based violence (WHO-VAW). |
|  | Nyongesa, Moses Kachama, et al (2022); Kenya | Depressive and anxiety symptoms (PHQ9 and GAD7) – within 2 weeks after the last session | Quality of life (FAHI) and perceived social support (Social provisions scale). |
|  | Dowrick, Christopher, et al (2022); UK | Anxiety and depressive symptoms at 3 months, measured using the HADS at 3 and 6 months | Subjective well-being (WHO-5), functional status (WHODAS), progress on identified problems (PSYCHLOPS), post-traumatic stress disorder (PCL-5), depressive disorder (PHQ-9) and service usage (Client Service Receipt Inventory (CSRI]) |
|  | Knefel, Matthias, et al (2022); Austria: | General health (GHQ-28) 1 week post intervention | Distress by Post-Migration Living Difficulties Checklist (PMLD), Complex PTSD symptoms (ITQ), quality of life (WHOQOL BREF), self-identified problems (PSYCHLOPS), and integration (IPL-12) |
|  | Zhang, Hong, et al (2020); China | Anxiety and depression symptoms, independently measured at 3 months with the Hospital Anxiety and Depression Scale | Psychological outcome profiles (PSYCHLOPS), measure of functioning (WHODAS 2) |
|  | Spaaij, Julia, et al (2022); Switzerland | Psychological distress (K10); health and disability across six dimensions (WHODAS). Qualitative evaluation of feasibility and acceptability was explored from several key informant groups | Symptoms of depression and anxiety (HSCL-25); Previous exposure to potentially traumatic events (PTE); post-migration stressors symptoms of PTSD (PMLDC), information on health and other service use. |
|  | Qi, Aili, Fatao Wang, and Tiwang Cao (2023); China | Social anxiety (Social Anxiety Scale for Children) | Parenting Stress Index-Short Form (PSI-SF) and Social Support Rate Scale |
|  | Bryant, Richard A., et al (2022); Australia | Severity of anxiety and depressive symptoms measured using the Hospital Anxiety and Depression Scale (HADS) | Worry (Generalized Anxiety  Disorder Scale [GAD-7]); Sleep difficulties (adapted version of the Sleep Impairment Index [SII]); Affect (Positive and  Negative Affect Schedule [PANAS]); Anhedonia (Pleasure Scale -36); pandemic-related worries (the 36-item COVID Stress Scale) |
|  | Dozio E., Dill AS. & Bizouerne C. (2023); Central African Republic | General wellbeing (WHO-5) | Posttraumatic Stress Disorder Checklist for DSM-5 (PCL-5); functioning (WHODAS 2.0); Psychological Outcome Profiles (PSYCHLOPS) |
|  | Mediavilla R et al. (2023); Spain | Depressive and anxiety symptoms (Patient Health Questionnaire-Anxiety and Depression Scale [PHQ-ADS]) | Depression symptoms (PHQ-9) and anxiety symptoms (GAD-7); PTSD symptoms  (eight-item version of the PTSD Checklist [PCL-5]) |
|  | Musotsi P et al. (2022); Iraq | Mental health distress using WHODAS 2.0, PSYCHLOPS and PHQ-9 |  |
|  | Tay, Alvin Kuowei, et al., 2020 | Post Traumatic Stress Disorder, Complex PTSD, Major Depressive Disorder, the 5 scales of the Adaptive Stress Index , and a measure of resilience. | Anxiety symptoms, and Persistent Complex Bereavement Disorder |
|  | Perera et al. (2022); Venezuela | Mental health and well-being measured by WHO-5, PSYCHLOPS, and WHOQOL-BREF |  |
|  | Rahman A. et al (2019) | Symptom score of anxiety and depression, (HADS); | PTSD symptoms (PCL-5), functional  Impairment (WHODAS 2), problems for which the person sought  help (PSYCHLOPS), perceived social support (MDSPSS), depressive disorders (PHQ-9) |
