# Supplementary material for: A scoping review of the literature on the application and usefulness of the Problem Management Plus (PM+) intervention around the world
Source: BJPsych Open. 2024 Apr 23;10(3):e91. doi: 10.1192/bjo.2024.55 (PMC11060090; doi:10.1192/bjo.2024.55)
Supplement: Mwangala et al. supplementary material 4 — Mwangala et al. supplementary material [file S2056472424000553sup004.docx]

**Supplementary table 4: PM+ clients, PM+ delivery formats and PM+ helpers, their training and supervision**

|  | **Study** | **PM+ clients** | **PM+ delivery format** | **PM+ helpers, their training and supervision** |
| --- | --- | --- | --- | --- |
|  | Acarturk, C., et al (2022); Turkey | Syrian refugees with psychological distress. | Group PM+ & ECAU: Five weekly sessions.  Face-to-face; groups had 8-10 people, separated by gender. | **Non-specialists**: Arabic-speaking facilitators who were peer refugees. Peer refugees were eligible to be facilitators of gPM+ if they had completed at least 12 years of education. Before the gPM+ delivery, peer refugees had to undergo an 8-day training. Facilitators received weekly local group supervision by certified PM+ trainers. |
|  | Akhtar, Aemal, et al (2021); Jordan | Syrian refugees with psychological distress. | Group PM+ & ECAU: Five weekly sessions of 120 minutes each.  Face-to-face; groups had 6-12 people, separated by gender. Each session was delivered by 2 facilitators. | **Non-specialists**: who held a bachelor degree in a psychology or a field related to health and have proficiency in Arabic. One local supervisor working within the camp provided weekly supervision. The facilitators received eight days of training in the delivery of the GroupPM+ intervention, as well as basic counselling and group facilitation skills. Following training, GroupPM+ facilitators were required to complete two practice cycles, as lead facilitator and co-facilitator, under close supervision. |
|  | Bryant, Richard A., et al (2022); Jordan | Syrian refugees aged ≥18 years screening positive for distress and impaired functioning | Group PM+; Five weekly sessions of 120 minutes each.  Face-face; groups had 8-to 10 people, separated by gender. Each session was delivered by 2 facilitators. | **Non-specialists:** a bachelor’s degree in social sciences or a related health discipline, proficient in Arabic, but had no prior experience in delivering psychosocial programs. The facilitators received 8 days of training in the delivery of gPM+. Following training, the gPM+ providers were required to complete 2 practice groups, as a lead facilitator and as a cofacilitator, under close supervision. A local supervisor who worked within the camp provided weekly supervision throughout the trial. |
|  | Bryant, Richard A., et al (2022); Jordan | Syrian refugees aged ≥18 years screening positive for distress and impaired functioning | Group PM+ & ECAU, Five weekly sessions of 120 minutes each.  Face-to-face; groups had 6-12 people, separated by, gender. Each session was delivered by 2 facilitators. | **Non-specialists:** a bachelor’s degree in social science or related discipline, spoke Arabic, but had limited to no prior experience in psychosocial programmes. Facilitators each received eight days of training that included group facilitation skills and gPM + delivery, followed by supervision during two practice cycles of gPM+. |
|  | de Graaff, Anne M., et al (2020); Netherlands | Adult Syrian refugees with elevated psychological distress | Individual PM+ & CAU,  Five face-to-face weekly sessions of 90 minutes each. | **Non-specialists**: Helpers had at least high school education, a background in social work, teaching or another related field, and sufficient Dutch or English-speaking ability. They received 8 days of training followed by weekly face-to-face group supervision by PM+ trainers/supervisors throughout the trial. Trainers/ supervisors were mental health care professionals who underwent 5-day training. |
|  | de Graaff, Anne M., et al (2023); Netherlands | Adult Syrian refugees with elevated psychological distress and reduced psychosocial functioning | Individual PM+ & CAU (with an option of in person or video call).  Five weekly sessions of 90 minutes each | **Non-specialists**: Helpers were Arabic (and Dutch or English) speaking Syrian refugees with at least high school education and (professional) background in education, social work or related field and a Certificate of Conduct. Helpers received an 8-day training on CMDs, basic counselling skills, delivery of intervention strategies and self-care, followed by a practice case. Helpers met weekly for group supervision by a PM+ supervisor. |
|  | Rahman, Atif, et al (2016); Pakistan | Adult primary care attendees with high levels of both psychological distress and functional impairment | Face-to-face Individual PM+.  5 weekly face-to-face sessions lasting 90 minutes each. | Non-specialists lay health workers with 12 to 16 years of education, with no previous clinical training or experience in counseling, social work, clinical psychology, or psychiatry. The master trainer (K.S.D.) conducted a 6-day training with local mental health specialists who in turn provided an 8-day training program to 9 lay health workers. Training of both supervisors and health workers was followed by clinical practice with 3 practice cases each under supervision. The lay health workers were supervised in 2 groups on a weekly basis (2 hours) by the in-country supervisors. |
|  | Khan, M. N., et al (2019); Pakistan | Women aged ≥18 years, referred for screening based on the judgment of their health workers | Group PM+  5 weekly face-to-face sessions lasting 120 minutes each. Each gPM+ had 6 clients. | **Non-specialists**: local female lay-helpers with 16 years of education (graduates) and with no formal training of or prior experience in mental health. The training and supervision of lay-helpers followed an apprenticeship model. Three non-specialist supervisors and two female lay-helpers received 6 days training by the Master Trainer. This was followed by four weeks of practice cases with weekly group supervision through Skype (2–3 h duration) by the 3 supervisors. Before delivering Group PM+ as part of the trial, lay-helpers completed competency assessments. |
|  | Hamdani, Syed Usman, et al (2020); Pakistan | Primary care attendees with high levels of psychological distress | Face-to-face Individual PM+  5 weekly face-to-face sessions lasting 90 minutes each. | **Non-specialists:** lay health workers (with 12–16 years of education). Training and supervision followed a cascade model. An international trainer trained local trainers during a 6-day training workshop. Training consisted of intervention delivery, training, and supervision skills. Local trainers cascaded the training to lay health workers in an 8-day training. Lay health workers were provided weekly supervision by local trainers/supervisors, who were in turn supervised monthly by the international trainer/supervisor via video conference for 2–3 h. |
|  | Hamdani, Syed Usman, et al (2021); Pakistan | Adult outpatient department attendees, referred for psychological support by psychiatrists | Face-to-face Individual PM+/TAU  5 weekly face-to-face sessions lasting 90 minutes each. | **Specialists**: a master’s degree (16 years of education) in psychology and received eight days training in PM+ by the master trainer followed by fortnightly supervision meetings with the master trainer. |
|  | Sangraula, M., et al (2020); Nepal | Adults with high levels of psychological distress and functional impairment | Group PM+  5 weekly face-to-face sessions lasting 2.5 to 3 hours each and separated by gender and with gender-matched facilitators  Each gPM+ had 6-8 clients. | **Non-specialists:** Community-based psychosocial workers (10 years of education, >25 years) were the service providers for the groups and are a cadre of psychosocial workers in Nepal that are trained through and work for NGOs. Volunteer local helpers supported facilitators by organising logistics and reminding participants about the sessions. Facilitators were trained for 20  Days. Group ‘helpers’ received a basic 2-day training on assisting facilitators : logistics and childcare |
|  | Jordans, Mark JD, et al (2021); Nepal | Adults with high levels of psychological distress and functional impairment in a disaster-prone setting | Group PM+  5 weekly face-to-face sessions lasting 2.5 hours each and separated by gender and with gender-matched facilitators | **Non-specialists:** had to be living in the communities where the project took place, not having received prior mental health training, having completed higher secondary school (equivalent to high school graduation), as well as based on interviews demonstrating adequate communication skills and motivation to serve members of their communities. Facilitators first received a 10-day training on foundational helping skills, followed by 10 days of Group PM+ facilitator training with subsequent supervised practice sessions. Face-to-face group supervision was provided weekly. |
|  | Dawson, Katie S., et al (2016); Kenya | women affected by urban adversity and gender-based violence | Face-to-face Individual PM+  5 weekly face-to-face sessions lasting 90 minutes each. | **Non-specialists:** Community health workers (CHWs) - have varied levels of education and do not receive any training or experience in mental health care. An eight-day training program was delivered by the master trainer, directly to the CHWs (N = 23) and three Kenyan psychologists who would provide supervision to the CHWs. The classroom training was followed by four weeks of practice cases (approximately three clients per CHW) under close supervision. CHWs were then required to pass competency assessments before offering PM+ to participants involved in the feasibility study. Supervision cascaded from a foreign intervention specialist to local experts, and onwards to CHWs. CHWs were supervised on a weekly basis by one of three local supervisors. |
|  | Bryant, Richard A., et al (2017); Kenya | women with a history of gender-based violence in urban in Nairobi Kenya | Face-to-face Individual PM+  5 weekly face-to-face sessions lasting 90 minutes each. | **Non-specialists:** 23 CHWs were engaged to provide PM+. The CHWs had 10 years’ school education and did not have prior training or experience in mental healthcare. The CHWs were provided with a 64-hour training program over 8 days. Two local supervisors who were experienced psychologists were also trained in PM+. Training covered knowledge of common mental health conditions, basic counselling delivery, PM+, and self-care strategies. CHWs also received a 1-day training in PFA to prepare them for managing people in crisis (e.g., ongoing violence) who required immediate attention and possible referral. Each CHW delivered PM+ to approximately 3 clients under local supervision, after which CHWs were assessed for competency based on the supervisor’s evaluation of mock interviews. During the trial, CHWs received 2 hours of weekly supervision by the local supervisor, who provided the supervision in 4 separate groups to the CHWs (5 CHWs per group). The local supervisors received 1.5 hours of weekly training and mentoring in supervision via Skype. |
|  | Nyongesa, Moses Kachama, et al (2022); Kenya | Young people living with HIV with mild-to-moderate symptoms of CMDs | Individual PM+ delivered over the phone.  10 weekly over-the-phone sessions lasting 45 minutes each. | **Non-specialists:** at least high school education but did not have previous mental health training. These lay helpers were identified from the community through advertisement of the study position followed by an interviewing process. A 3-week training programme (including 10 days of in-field practice) was delivered by a clinical psychologist trained on PM+, who was certified as a trainer of trainers. Four lay helpers (two females, two males; age range 26–35 years) and an additional two master-level staff at CGMR-C, who would provide supervision to the lay helpers, were trained. |
|  | Dowrick, Christopher, et al (2022); UK | distressed and functionally impaired asylum seekers and refugees | Individual and Group PM+.  5 weekly sessions of about 90 minutes each (for Individual PM+) or 120 minutes (for the group version) | **Non-specialists:** trained lay therapists with lived experience of the asylum process. 2 wellbeing mentors and supervisors received 5 days of training from PM+ master trainers. Lay therapists received 8 days of training followed by practice cases and competency assessment. |
|  | Knefel, Matthias, et al (2022); Austria: | Afghan asylum seekers or refugees which were seeking help for mental health problems | Face-to-face Individual PM+/TAU  6 weekly face-to-face sessions lasting 90 minutes each. | **Specialists**: clinical psychologists - The psychologists were trained by two WHO PM+ master trainers. |
|  | Zhang, Hong, et al (2020); China | Cases of multiple myeloma who presented depressive symptoms | Face-to-face individual PM+  5 weekly sessions lasting 90 minutes each.  Relatives and caregivers received training for transitional care skills | **Non-specialists:** nurses. Relatives and caregivers received training for transitional care skills. |
|  | Spaaij, Julia, et al (2022); Switzerland | Syrian refugees and asylum seekers experiencing elevated levels of psychological distress | Face-to-face Individual PM+  5 weekly face-to-face sessions lasting 90 minutes each. | **Non-specialists:** Thirteen non-specialist ‘helpers’ and eight lay outcome assessors were recruited. All helpers and outcome assessors were Syrians and fluent in Arabic and German or English. All of them had a diploma of higher education. Helpers participated in an eight-day training course conducted by one PM+ master trainer and one PM+ trainer, both Arabic speaking, and the research team in line with WHO training criteria. Before the intervention, each helper administered at least one practice case under supervision to become familiar with the intervention and all of its procedures. All helpers received continuous supervision (at the beginning on a weekly basis and later approximately once per month) by an experienced Arabic speaking clinical psychologist/psychotherapist and PM+ master trainer with extensive knowledge of the PM+ intervention. |
|  | Qi, Aili, Fatao Wang, and Tiwang Cao (2023); China | parents of children with ASD | Individual PM+ & Tai Chi training in addition to routine health education.  PM+ intervention: the course was conducted in the form of live streaming regularly 14 times in 7 weeks, 2 times each week, 40 minutes each time | Not reported |
|  | Bryant Richard A et al (2022); Australia | Adults in Australia distressed by COVID-19 | adapted Group PM+ delivered to groups of four participants on a videoconferencing platform. It consisted of 6 × 60-min sessions conducted over a period of 8 weeks | The intervention comprised 6 × 60-min sessions that were delivered to groups of four participants on a videoconferencing platform by **a Masters or Doctoral level clinical psychologist** The facilitators were trained by Master trainer over 8 days, and each led a full practice group program under supervision by master trainer |
|  | Mediavilla et al., 2023; Spain | HealthCare Workers employed by the Department of Health with psychological distress | Participants received eCAU; then offered the stepped-care programme (comprised two scalable psychological interventions: a guided stress management course based on the SH+ booklet called Doing What Matters in Times of Stress (DWM) and PM+); Both interventions had an online format, each took 5–6 weeks to be delivered | The intervention providers **were mental health providers (psychiatry**, clinical psychology, and mental health nursing trainees) who received specific preparation. Attendance of weekly supervision sessions by intervention providers while the trial was ongoing (~30 hours); The trainers/supervisors were psychiatrists and clinical psychologists instructed by the intervention developers. |
|  | Dozio, Dill & Bizouerne, 2023; Central African Republic | Internally displaced persons | PM+ adapted for group use (max 10 people per group), 5 weekly sessions of approximately 2hours each. | Each group faciliated by two psychosocial workers who received a 2-week initial training (including group facilitation techniques). continuous supervision of the trained psychosocial  workers by an expert psychologist. |
|  | Perera et al., 2022; Venezuela | Venezuelan refugees and migrants and Colombian returnees | Individual PM+  5 weekly sessions each 90 mins | 4-day training of PM+ to Colombian Red Cross volunteers and Supervisors. All intervention providers had previous training in PFA. Two Colombian Red Cross volunteers and two supervisors were trained over the course of four days to deliver PMþ in July 2018. A second group of nine Colombian Red Cross volunteers were also trained in a second four-day training, held in November 2018. |
|  | Musotsi, Protus, et al_2022 Iraq | IDPs, returnees and host community adults with emotional distress and/or functional impairment | Individual PM+ was delivered over five weeks, with each  weekly session lasting 90 minutes. | Lay counsellors received 3 weeks of training. Four days were used for training on psychological first aid (PFA), enhanced with basic psychosocial support skills and 8 days for PM+ training in the classroom and the field. Also received positive parenting training for 2 days. Training was delivered by a Senior Technical Advisor for MHPSS, experienced in all PM+ strategies, with the help of the organisation’s in-country trained MHPSS specialist.  Lay counsellors underwent supervised in-field practice, offering 5 sessions of PM+ to three clients with less severe presentations. Lay counsellors received weekly clinical supervision from trained professional psychologists who were Trainer of Trainers in PM+, DWM and PFA. The supervisors (psychologists) received biweekly supervision from the organisation’s Global Senior Technical MHPSS Advisor. |
|  | Rahman et al., 2019; Pakistan | Adult women aged 18-60 years in a conflict area. | The group PM+  Intervention consisted of five group sessions per week, with approximately six to eight participants per group, each session lasting for approximately 2 h (excluding breaks) | **The therapists, called facilitators**, were local graduates with bachelor’s degrees without mental health-care experience. The facilitators received 7 days of intervention training by a master trainer (KSD) and were supported by three in-country supervisors based in Islamabad, Pakistan  (PA, HN, and AM. To assess competency, all facilitators delivered one practice group each at an accelerated rate (five sessions in 2 weeks) with participants living outside the trial area and under intensive supervision (10 h supervision over 2 weeks). |
|  | Tay, Alvin Kuowei, et al., 2020 | Rohingya, Chin, and Kachin refugees living in Malaysia | PM+ (here referred to as Cognitive Behavioural Therapy - CBT) was evaluated as a comparator intervention to a primary intervention called Integrative Adapt Therapy - IAT). CBT included 6 strategies – drawn from WHO PM+ with an additional component of cognitive reappraisal.  The strategies were delivered sequentially over 6-weekly sessions, each session 45 minutes. | CBT was delivered by trained lay counsellors. Eight days were devoted to training in IAT and the same period for CBT, the 2 programs being taught separately, using 2 manuals—one for IAT and one drawing on WHO PM+. Half of the counsellors received the 2 sets of training in one sequence, the other in the reverse order. In both trainings provided by the research team. Trainees then progressed to 8 weeks of field practice with members of the respective communities meeting selection criteria, followed by 6 months of supervised implementation prior to commencement of the trial. Each counsellor was assessed for their competency in basic counselling and in applying the treatment strategies specific to both CBT and IAT. |
