# Supplementary material for: A scoping review of the literature on the application and usefulness of the Problem Management Plus (PM+) intervention around the world
Source: BJPsych Open. 2024 Apr 23;10(3):e91. doi: 10.1192/bjo.2024.55 (PMC11060090; doi:10.1192/bjo.2024.55)
Supplement: Mwangala et al. supplementary material 5 — Mwangala et al. supplementary material [file S2056472424000553sup005.docx]

**Supplementary Table 5: PM+ Effectiveness analysis**

| **Study** | **Effect sizes analyses across different covariates** | | | |
| --- | --- | --- | --- | --- |
|  | **Study outcomes *(bolded are primary outcomes) only significant values are given*** | **Study population** | **Delivery modality** | **Gender of PM+ clients** |
| 1. Bryant, R. A., et al. (2022). | **Anxiety = 0.4;**  **Depression = 0.4;**  Worry = 0.3;  Anhedonia = -0.4;  Positive mood of PANAS = -0.6;  Negative mood of PANAS = 0.3;  COVID-19 related fears = 0.3;  Xenophobic fears = 0.3;  Contamination fears = 0.3; | Adults distressed by COVID-19 in the community (84% women) | Group-based PM+ delivered via videoconferencing | Results not aggregated by gender |
| 1. Mediavilla, R., et al. (2023) | **Anxiety/depression = 0.8**;  Depression = 0.8;  Anxiety = 0.6;  Posttraumatic stress = 0.5; | Healthcare workers with psychological distress during the COVID pandemic (86% women) | Remote delivery (weekly phone-based or message-based) | Results not aggregated by gender |
| 1. Bryant, R. A., et al. (2017). | **Psychological distress = 0.6**;  functional impairment = 0.3  Posttraumatic stress = 0.2  Personally identified problems = 0.7;  Stressful life events =  Health service utilization = | Women who had experienced gender-based violence | Individual PM+ delivered face to face | All participants were women. |
| 1. Rahman, A., et al. (2016). | **Anxiety/depression symptoms** = 0.8;  posttraumatic stress = 0.6;  functional impairment = 0.7;  personally identified problems = 0.3;  depressive symptoms = 0.7 | Adult primary care attendees with high levels of both psychological distress and functional impairment in a conflict affected area (79% women) | Individual PM+ delivered face to face | Results not aggregated by gender |
| 1. De Graaff, A. M., et al. (2020). | **Depression/anxiety = 0.6**;  Functional impairment = 0.7  Posttraumatic stress = 0.7  Self-identified problems = 0.8 | Syrian refugees with elevated psychological distress (60% women) | Individual-based PM+ delivered face-to-face | Study did not evaluate gender-specific effects |
| 1. Study | **Study outcomes *(bolded are primary outcomes) only significant values are given*** | **Study population** | **Delivery modality** | **Gender of PM+ clients** |
| 1. De Graaff, A. M., et al. (2023). | **Depression/anxiety = 0.4**;  Anxiety = 0.3;  Depression = 0.4;  Posttraumatic stress = 0.4;  Personally identified problems = 0.3 | Syrian refugees with elevated psychological distress and reduced psychosocial functioning (38% women) | Individual PM+ (with option of in person or video call) | Study did not evaluate gender-specific effects |
| 1. Bryant, R. A., et al. (2022). | **Depression = 0.4**;  Personally identified problems = 0.5;  Parenting behaviour = 0.7 | Syrian refugees (73% women) | Group PM+ delivered face-to-face | Results not aggregated by gender |
| 1. Jordans, M. J., et al. (2021). | **Psychological distress** = 0.2 | Adults with high levels of psychological distress and functional impairment in a disaster-prone setting (82% women) | Group PM+ delivered face-to-face | Study did not evaluate gender-specific effects |
| 1. Rahman, A., et al. (2019). | **Depression/anxiety = 0.6**;  Anxiety = 0.6;  Depression = 0.5;  Functional impairment = 0.4;  Personally identified problems = 0.4 | Adult women in a post-conflict setting | Group PM+ delivered face-to-face | All participants were women. |
| 1. Bryant, R. A., et al. (2022). | Positive parenting = -0.4 | Syrian refugees screening positive for distress and impaired functioning (70% women) | Group PM+ delivered face-to-face | Study did not evaluate gender-specific effects |
| 1. Tay, Alvin Kuowei, et al., 2020 | **Post-traumatic stress disorder (PTSD) = 0.9**  **Complex PTSD = 1.02**  **Major depressive disorder = 1.1**  **Resilience** = 0.2  Anxiety symptoms = 1.2  Persistent Complex Bereavement Disorder = 0.3 | Rohingya, Chin, and Kachin refugees living in Malaysia (72% men) | Individual CBT delivered face-to-face | Study did not evaluate gender-specific effects |
|  |  |  |  |  |
